# What is the role of food consumption in the relationships between sleep duration, sleep quality, and cognitive function? A study among Chinese older adults

**DOI:** 10.1186/s12877-026-07037-1

**Published:** 2026-02-27

**Authors:** Hao Wu, Xiaona He, Yu Cao, Huiting Chen, Wei Gao

**Affiliations:** 1 Department of Epidemiology & Health Statistics, School of Public Health, Jiangxi Medical College, Nanchang University, Nanchang, Jiangxi China; 2 Jiangxi Provincial Key Laboratory of Disease Prevention and Public Health, Nanchang University, Nanchang, China

**Keywords:** Sleep duration, Sleep quality, Food consumption, Cognitive function, Mediating effect, Chinese older adults

## Abstract

**Background:**

As people age, declines in physiological functions can lead to changes in sleep and dietary patterns, potentially influencing cognitive function. However, longitudinal evidence examining how food consumption mediates the relationship between sleep parameters and cognitive function in Chinese older adults remains limited. Examining these longitudinal trajectories, beyond single-timepoint measurements, is crucial for understanding their dynamic nature and impact on cognitive function. Therefore, this study aimed to assess the longitudinal trajectories of sleep and food consumption in older adults, and to examine the mediating role of food consumption patterns in the sleep-cognition relationship.

**Methods:**

The sample population included 8724 older adults who derived from waves 4 to 7 (2005 to 2014) in the Chinese Longitudinal Healthy Longevity Survey (CLHLS). Self-reported sleep duration, sleep quality, and food consumption were evaluated in face-to-face interviews. The main outcomes were global cognitive composite z scores. Group-based trajectory modeling (GBTM) identified distinct long-term change patterns of sleep duration, sleep quality and food consumption, and generalized estimating equations (GEE) evaluated their prospective associations with global cognitive scores. Additionally, structural equation modeling (SEM) was performed to assess the mediating role of food consumption in the relationships between sleep duration, sleep quality, and cognitive function.

**Results:**

Food consumption mediated the associations between sleep duration, sleep quality, and cognitive function in older adults. After adjusting for confounders, decreasing low fruit and vegetable consumption mediated a negative impact on cognition, with both shortened (0.024, 95%CI: 0.003 to 0.048) and excessive sleep duration (0.076, 95%CI: 0.040 to 0.117) linked to decreasing low fruit and vegetable consumption. A rapid decline in meat, fish, egg, and bean consumption also mediated the negative effect of shorter sleep duration on cognition (mediation effects: -0.009, 95%CI: -0.014 to -0.005), whereas increased milk consumption acted as a complete mediator, positively influencing cognition. No mediating effect was observed for nuts consumption. Furthermore, stable, high-quality sleep promoted increased consumption of fruit and vegetable (0.039, 95%CI: 0.020 to 0.058), meat, fish, egg, and bean (-0.011, 95%CI: -0.133 to -0.088), milk (0.022, 95%CI: 0.001 to 0.043), and nuts (0.043, 95%CI: 0.021 to 0.065), all positively associated with cognitive levels.

**Conclusion:**

This study illustrates that food consumption can improve the decline in cognitive levels in older adults that may be undermined by unhealthy sleep habits. Our research findings are of great importance to both clinical practice and public health policy.

**Supplementary Information:**

The online version contains supplementary material available at 10.1186/s12877-026-07037-1.

## Introduction

Aging is the primary contributor to a broad spectrum of chronic disorders all associated with poor quality of life in older adults [1, 2]. By 2050, it is expected that there will be 1.4 billion Chinese, with 365 million aged 65+, a number representing 26.1% of the country’s total population [1, 3]. China shares some of the economic and social challenges faced by other countries with rapidly aging populations [4]. Sleep architecture and circadian patterns change as people age [5], and the overall sleep duration typically decreases in older adults. The National Sleep Foundation recommends that the appropriate sleep duration of older adults is 7 to 8 h as normal sleep [6]. But many older persons continually deprive themselves of adequate sleep [7]. In addition, aging is also associated with a decline in cognitive skills [8–10]. A faster rate of cognitive decline leads to not only premature cognitive impairment and dementia but also a shorter life span [11–13]. Consequently, the number of older adults with cognitive impairment and dementia is increasing rapidly.

More importantly, disruption of sleep quality and reduction in sleep quantity lead to excessive neuronal activity [14]. For example, sleep apnea and chronic insomnia can lead to oxidative stress and neuronal damage. These changes predispose and culminate in the development of cognitive impairment. Most evidence examined the relationships between sleep duration, sleep quality, and cognitive decline in older people using large-scale samples [15–17]. Findings from epidemiological studies indicated that an inverted U-shaped association between sleep duration and cognitive decline was observed [15, 18], indicating that insufficient or excessive sleep duration was significantly associated with faster cognitive decline. Whereas some studies suggested long [19] or short sleep phenotype [20, 21] was related to the risk of cognitive decline. Additionally, changes in sleep duration over time were independently associated with cognitive decline and may be a marker of cognitive decline [21]. Furthermore, poor sleep quality increased the risk of developing cognitive impairment and dementia [16, 22, 23].

Multidisciplinary interventions attempt to reduce the adverse effects of sleep-related issues on cognitive decline, with dietary interventions potentially acting as a key mediator in this pathway. Crucially, impaired sleep can negatively influence dietary patterns, potentially accelerating cognitive decline through suboptimal nutrition. Previous research suggests that poor sleep quality or short sleep duration was associated with increased consumption of energy-dense, nutrient-poor foods, reduced intake of fruits and vegetables, and altered appetite regulation [24–27]. This sleep-induced shift towards less healthy diets might subsequently compromise cognitive health. One research observed insufficient sleep is associated with reduced consumption of neuroprotective foods (e.g., vegetables, fruits, fish, and fiber-rich sources) and disrupted meal timing [17], which may independently impair cognitive resilience. Furthermore, the healthy diet may have a positive impact on cognitive health is well-supported by numerous studies [28–30]. As evidenced by the fact that high vegetable but not fruit consumption may be associated with a slower rate of cognitive decline with older age [31]. Therefore, dietary interventions may serve as a modifiable factor connecting sleep problems and cognitive decline in older adults.

While existing evidence had largely relied on cross-sectional assessments or single baseline measurements of sleep and food consumption, these approaches failed to capture the dynamic nature over time. Both sleep patterns and dietary habits were not static but were known to fluctuate and evolve, particularly in older adults, due to physiological changes, retirement, and evolving health status. Consequently, investigating the long-term trajectories—beyond mere baseline levels—was critical. However, the relationships between sleep duration, sleep quality, food consumption, and cognitive levels are less studied among Chinese older adults. Accordingly, this study also hypothesized that food consumption, as an intermediate mechanism, impacts the association between sleep duration, sleep quality, and cognitive levels in Chinese older adults.

## Methods

### Study design and sample

**Fig. 1:**
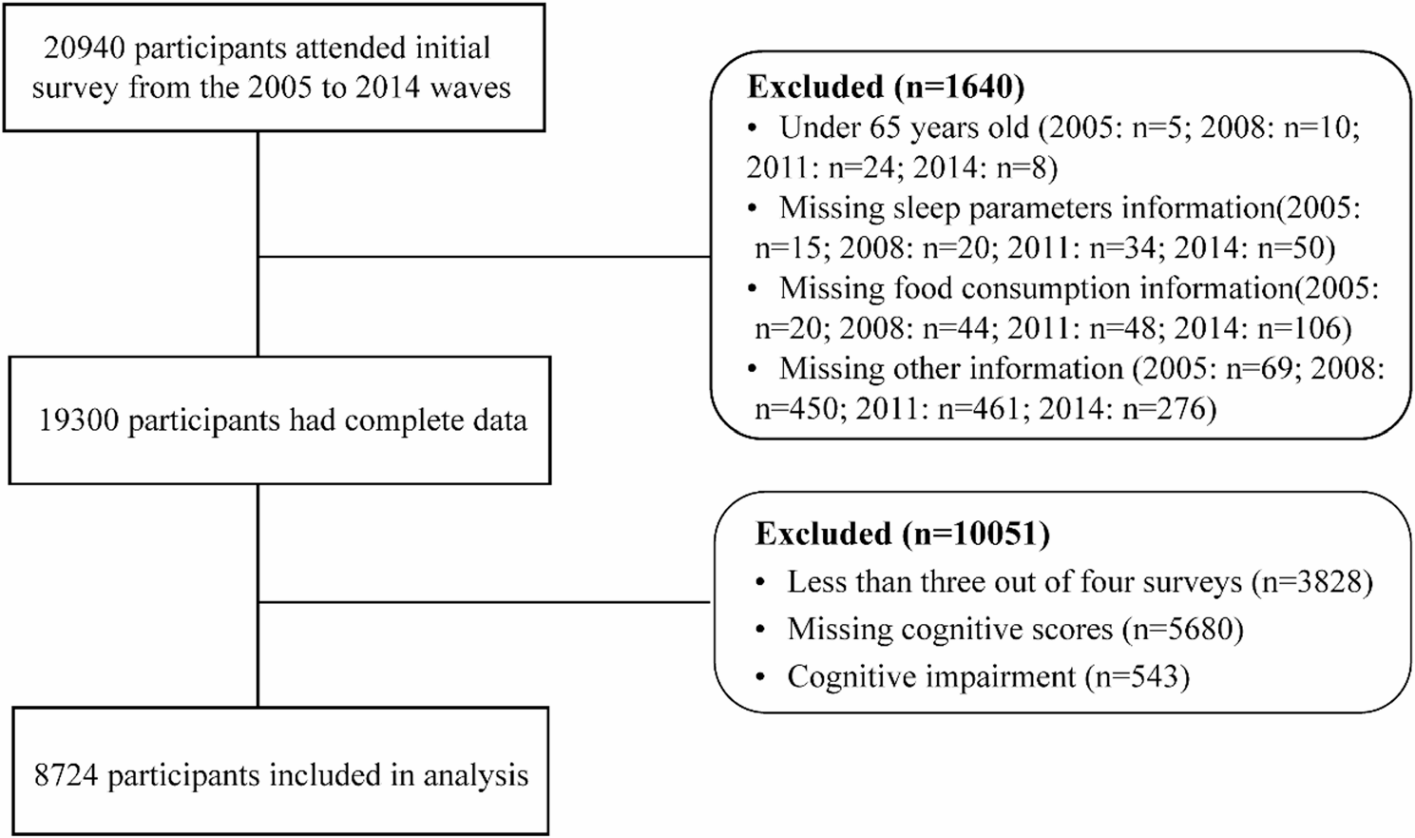
Flowchart of Participant Selection for the Study

The Chinese Longitudinal Healthy Longevity Survey (CLHLS) and interdisciplinary research conducted by Peking University, conducted a baseline survey in 1998 and 8 follow-up surveys from 1998 to 2018 in 23 provinces and autonomous regions, with 20,940 household visits. The survey collected detailed data on the basic demographics, physical function, health status, quality of life, medical costs, and long-term care needs among older adults before death. Given that sleep-related parameters were first assessed in 2005, we used data from the 2005 to 2014 waves of the CLHLS. We identified 8724 participants who attended at least 3 of the 4 follow-up surveys, excluding subjects with incomplete and ineligible information (Fig. 1).

The Research Ethics Committees of Peking University and Duke University granted approval for the Protection of Human Subjects for the Chinese Longitudinal Healthy Longevity Survey, including a collection of the data used for the present study. The survey respondents gave informed consent before participation. Additional details regarding the research design and data collection of the CLHLS have been described elsewhere [32].

## Measures

### Sleep duration and sleep quality

Baseline sleep parameters were self-reported without any given categories in face-to-face interviews. Sleep duration was ascertained using the question: “How many hours do you sleep normally every day including napping?”, with respondents giving an integer number. Sleep quality was asked as follows: “How do you describe your sleep quality recently?” The five response choices were very good, good, fair, bad, and very bad. The questions on sleep duration and quality were specific to the past week, or at most two weeks. Previous research had extensively employed a single-item self-report questionnaire to assess sleep quality, whereby a strong correlation with more comprehensive multi-item scales designed to measure sleep quality [33].

We employed Group-Based Trajectory Model (GBTM) to identify and analyze trajectories of sleep quality and sleep duration from 2005 to 2014. The optimal trajectory model was selected by the minimum Bayesian Information Criterion (BIC), minimum Akaike Information Criterion (AIC), minimum logarithmic likelihood (ll) and maximum entropy. In addition, the group member prediction probability (PPGM, > 5%) was used as additional statistical indicators [34]. Using GBTM, it was found that as the number of trajectories increased, the absolute values of the BIC gradually decreased (see Additional File 2, Table 2.1). The selection of the optimal trajectory model was based on the minimum BIC, AIC, and LL values, along with the maximum Entropy. Accordingly, the participants were divided into 3 groups according to sleep duration for the analyses: “increasing and long sleep duration”, “stable adequate sleep duration”, and “decreasing and short sleep duration”, with stable adequate sleep duration as the reference group (see Additional File 2, Fig. 2.1). We categorized sleep quality into increasing low sleep quality, stable high sleep quality, and decreasing sleep quality, with decreasing sleep quality as the reference group (see Additional File 2, Fig. 2.2).

### Food consumption trajectories

A simplified food frequency questionnaire (FFQ) was utilized to evaluate dietary habits, collecting data through self-reports from participants. In the original questionnaire, the frequency of eight food consumption was recorded through face-to-face interviews. Specifically, fruit and vegetable consumption were followed by every day or almost every day; quite often; occasionally; rarely, or never. Each category is assigned a value of 3, 2, 1, or 0, respectively. The total score of fruit and vegetable consumption ranged from 0 to 6, and a higher score indicated more fruit and vegetable consumption. The remaining 6 food groups including meat, fish, egg, bean, milk, and nuts were categorized as almost every day; not every day, but at least once per week; not every week, but at least once per month; not every month, but occasionally; rarely, or never. Each category is assigned a value of 4, 3, 2, 1, or 0, respectively. Accordingly, the total score of meat, fish, egg, and bean consumption ranged from 0 to 16, and the score of milk and nuts consumption each ranged from 0 to 4, respectively.

The GBTM was conducted to identify and analyze trajectories of food consumption from 2005 to 2014. The selection of the optimal trajectory model was based on the minimum BIC, AIC, and LL values, along with the maximum Entropy. Based on these considerations, a three-trajectory model (including two quadratic trajectories and one cubic trajectory) was identified as the best-fitting model. The optimal trajectories of fruit and vegetable consumption included “stable high fruit and vegetable consumption”, “stable moderate fruit and vegetable consumption”, and “decreasing low fruit and vegetable consumption” (see Additional File 2, Fig. 2.3). The control group was defined as the “stable moderate fruit and vegetable consumption” group. Similarly, the best-fitting trajectory of meat, fish, egg, and bean consumption identified two distinct patterns: slowly decreasing and rapidly decreasing meat, fish, egg, and bean consumption (see Additional File 2, Fig. 2.4). The “slowly decreasing meat, fish, egg, and bean consumption” group served as the referencing group. Milk consumption was classified into “never drink milk” and “increasing milk consumption”, while nuts consumption was categorized into “never eat nuts” and “increasing nuts consumption” (see Additional File 2, Fig. 2.5 and Fig. 2.6). The control group was defined as individuals who never drank milk or never ate nuts.

### Cognitive function

The Chinese version of the Mini-Mental State Examination (MMSE) was used to assess cognitive status. Cognitive assessment was conducted in the 2005 to 2014 waves and included the five aspects of orientation, registration, attention and calculation, recall, and language with scores ranging from 0 to 23. The higher values indicated better performance. We used standardization methods used in multiple studies to calculate z scores for cognitive function [15]. The cognitive function data was analyzed by generating z scores to compare across tests. Each test score was standardized by subtracting the mean and dividing by the standard deviation (SD) to the baseline score. The z score for global cognitive scores was calculated by averaging the z scores for the five tests and re-standardizing to baseline according to the mean and SD of the baseline global cognitive z scores. To reduce the influence of cognitive impairment on sleep duration, sleep quality, and food consumption, we excluded participants scoring 1.5 SDs or more below the mean for all waves, a cutoff that had been used to identify cognitive impairment [35].

### Covariates

The associations between sleep duration, sleep quality, food consumption, and cognitive scores in the analyses may be influenced by covariates. The following covariates were selected based on prior literature: sociodemographic information (age, sex, district, residence type, main occupation before the age of 60, current marriage status), health-related behavior (smoking, drinking, exercising status, and the frequency of social activities), health status (chronic diseases, systolic and diastolic blood pressure, body mass index (BMI), heart rate, and depression). Additional information was provided in Additional File 1.

### Statistical analysis

The results are displayed as number (percentage) for discrete variables and as mean (SD) or median (interquartile range [IQR]) for continuous variables. Descriptive analysis was conducted to compare the characteristics of the study population stratified by age (65–74 years, 75–84 years, and ≥ 85 years), sleep quality, and sleep duration. Statistical differences were assessed by χ^2^ test and 1-way analysis of variance (ANOVA) with Šídák multiple comparison test used for post hoc analysis for continuous variables.

The Group-based Trajectory Modeling (GBTM) was employed to model the long-term change patterns of sleep parameters and food consumption. Based on the latent class model, the GBTM attempted to divide the observed individual trajectories into several potential groups, each with a similar pattern of trajectories, and used maximum likelihood estimation to classify individuals with similar developmental trajectories [36, 37]. This approach allowed researchers to uncover potential group structures hidden in the data without knowing the group membership beforehand. We used the censored normal (CNORM) distribution for trajectory model fitting. For each outcome variable—including sleep duration, sleep quality, and the four food categories (fruits and vegetables; meat, fish, egg, and bean; milk; nuts)—we fitted a series of models specifying one to four trajectory groups. We tested both linear (first-order) and quadratic (second-order) polynomial functions to determine the shape of each trajectory. The optimal trajectory model was selected by the minimum Bayesian Information Criterion (BIC), minimum Akaike Information Criterion (AIC), minimum logarithmic likelihood (ll) and maximum entropy. In addition, the group member prediction probability (PPGM, > 5%) was used as additional statistical indicators [34]. After the optimal models were selected, each participant was assigned to the trajectory group for which they had the highest posterior probability of membership. These trajectory group assignments were used as the primary exposure variables in subsequent generalized estimating equation (GEE) models to examine their associations with cognitive function after adjusting for all covariates.

Structural equation modeling (SEM) was performed to construct the relationship between sleep duration, sleep quality, food consumption, and global cognitive z scores. We tested the indirect association of food consumption as a mediator, the global cognitive z scores were used as the outcome, to explain the association of sleep duration, and sleep quality with cognitive function. Optimal models were chosen by applying the following criteria: (1) comparative fit index (CFI) > 0.95; (2) Tucker-Lewis index (TLI) > 0.95; (3) root mean square error of approximation (RMSEA) < 0.06; (4) standardized root mean square residual (SRMR) < 0.06 [38, 39]. In the GEE and mediation models, sleep duration and sleep quality were included as simultaneous predictors to estimate their independent associations with cognitive function and food consumption trajectories. All statistical tests were 2-sided, and statistical significance was defined as *P* < 0.05. Analyses were performed with R version 4.3.2 (R Foundation, Vienna, Austria) and Mplus version 8.10 (Muthén & Muthén).

## Results

### Baseline characteristics

The flowchart of participant selection for this study is in Fig. 1. People experiencing decreased and short sleep duration were more likely to increase their intake of fruit, vegetable, milk, and nuts, and most likely to decrease their consumption of meat, fish, egg, and bean. Conversely, among those with increased and long sleep duration, reducing consumption of fruit and vegetable was the most common change. The cohort exhibiting stable sleep quality demonstrated consistently high sleep quality scores, elevated consumption of vegetable and fruit, and a more gradual reduction in the consumption of meat, fish, egg, and bean. Furthermore, this group exhibited the highest prevalence of individuals increasing their consumption of milk and nuts. Conversely, the cohort experiencing declining sleep quality was characterized by the highest proportion of individuals from milk and nuts consumption. Although the cohort reporting improved sleep quality exhibited a modest upward trend in sleep quality scores, overall scores remained comparatively low. This group also demonstrated moderate or declining trends in vegetable and fruit consumption and exhibited the highest prevalence of individuals reporting a pronounced decrease in the consumption of meat, fish, egg, and bean. The demographic data of the participants stratified by sleep duration (Table 1), sleep quality (see Additional File 2, Table 2.2), and age groups can be found (see Additional File 2, Table 2.3).

**Table 1:** Baseline characteristics of participants in the study (Stratified by sleep Duration)

Characteristic	Total (*n* = 8724)	Participants, No. (%)					
		Sleep duration trajectories per night, h					
		Increasing and long sleep duration (*n* = 280)	Stable adequate sleep duration (*n* = 6554)	Decreasing and short sleep duration (*n* = 1890)	Statistical test	*p*-value	*p*-value for post hoc test ^a^
Age, mean (SD), y	79.31(8.66)	86.53(9.68)	79.42(8.78)	77.86(7.42)	1-Way ANOVA	< 0.001	< 0.001 ^b, c, d^
Sex							
Female	4334(49.68)	123(43.93)	3439(52.47)	772(40.85)	χ^2^	< 0.001	NA
Male	4390(50.32)	157(56.07)	3115(47.53)	1118(59.15)			
District							
Eastern China	4984(57.13)	181(64.64)	3833(58.48)	970(51.32)	χ^2^	< 0.001	NA
Central China	2253(25.83)	79(28.21)	1660(25.33)	514(27.20)			
Western China	1487(17.04)	20(7.14)	1061(16.19)	406(21.48)			
Marital status							
Married	4483(51.39)	83(29.64)	3393(51.77)	1007(53.28)	χ^2^	< 0.001	NA
Divorced	32(0.37)	1(0.36)	22(0.34)	9(0.48)			
Widowed	4135(47.4)	196(70.00)	3081(47.01)	858(45.40)			
Never married	74(0.85)	0(0.00)	58(0.88)	16(0.84)			
Residence type							
City	1425(16.33)	43(15.36)	1036(15.81)	346(18.31)	χ^2^	0.064	NA
Town	2552(29.25)	77(27.50)	1953(29.80)	522(27.62)			
Rural	4747(54.41)	160(57.14)	3565(54.39)	1022(54.07)			
Main occupation before 60 y							
Non-manual workers	888(10.18)	6(2.14)	701(10.70)	181(9.58)	χ^2^	< 0.001	NA
Manual workers	6992(80.15)	243(86.79)	5205(79.41)	1544(81.69)			
Other	844(9.67)	31(11.07)	648(9.89)	165(8.73)			
Smoking status							
Present	1967(22.55)	56(20.00)	1516(23.13)	395(20.90)	χ^2^	< 0.001	NA
Previous	1529(17.53)	55(19.64)	1191(18.17)	283(14.97)			
None	5228(59.93)	169(60.36)	3847(58.70)	1212(64.13)			
Drinking status							
Present	1889(21.65)	58(20.71)	1506(22.98)	325(17.20)	χ^2^	< 0.001	NA
Previous	1323(15.17)	42(15.00)	1002(15.29)	279(14.76)			
None	5512(63.18)	180(64.29)	4046(61.73)	1286(68.04)			
Exercising status							
Present	3489(39.99)	107(38.21)	2599(39.65)	783(41.43)	χ^2^	0.247	NA
Previous	978(11.21)	28(10.00)	760(11.60)	190(10.05)			
None	4257(48.8)	145(51.79)	3195(48.75)	917(48.52)			
Social activities							
Yes	1690(19.37)	33(11.79)	1302(19.87)	355(18.78)	χ^2^	0.003	NA
No	7034(80.63)	247(88.21)	5252(80.13)	1535(81.22)			
Sleep quality							
Increasing low sleep quality	980(11.23)	0(0.00)	368(5.61)	612(32.38)	χ^2^	< 0.001	NA
Stable high sleep quality	6661(76.35)	274(97.86)	5656(86.30)	731(38.68)			
Decreasing sleep quality	1083(12.42)	6(2.14)	530(8.09)	547(28.94)			
Fruit and vegetable consumption							
Stable high fruit and vegetable consumption	1871(21.45)	52(18.57)	1407(21.47)	412(21.80)	χ^2^	< 0.001	NA
Stable moderate fruit and vegetable consumption	6611(75.78)	199(71.07)	4998(76.26)	1414(74.81)			
Decreasing low fruit and vegetable consumption	242(2.77)	29(10.36)	149(2.27)	64(3.39)			
Meat, fish, egg and bean consumption							
Slowly decreasing	6411(73.49)	196(70.00)	4977(75.94)	1238(65.50)	χ^2^	< 0.001	NA
Rapidly decreasing	2313(26.51)	84(30.00)	1577(24.06)	652(34.50)			
Milk consumption							
Never drink milk	2146(24.6)	65(23.21)	1675(25.56)	406(21.48)	χ^2^	0.001	NA
Increasing milk consumption	6578(75.4)	215(76.79)	4879(74.44)	1484(78.52)			
Nuts consumption							
Never eat nuts	2382(27.3)	113(40.36)	1779(27.14)	490(25.93)	χ^2^	< 0.001	NA
Increasing nuts consumption	6342(72.7)	167(59.64)	4775(72.86)	1400(74.07)			
Number of chronic diseases							
0	3733(42.79)	135(48.22)	2938(44.83)	660(34.92)	χ^2^	< 0.001	NA
1	2360(27.05)	69(24.64)	1754(26.76)	537(28.41)			
≥2	2631(30.16)	76(27.14)	1862(28.41)	693(36.67)			
Blood pressure, mean (SD), mm Hg							
Systolic	135.12(23.03)	136.15(21.75)	134.86(22.45)	135.84(25.10)	1-Way ANOVA	0.202	0.737, ^b^ 0.995, ^c^ 0.285 ^d^
Diastolic	80.97(17.31)	81.95(13.35)	80.81(16.69)	81.39(19.77)		0.280	0.630, ^b^ 0.942, ^c^ 0.492 ^d^
Global Cognitive scores, MMSE, mean (SD)	20.32(3.24)	19.34(3.84)	20.34(3.25)	20.38(3.08)	1-Way ANOVA	< 0.001	< 0.001 ^b, c^ 0.926 ^d^
Depression scores, mean (SD)	6.48(3.49)	6.09(3.53)	6.34(3.43)	7.01(3.65)	1-Way ANOVA	< 0.001	0.545, ^b^ <0.001 ^c, d^
Heart rate, mean (SD), beats/min	74.01(15.15)	74.43(9.86)	73.91(15.08)	74.29(16.03)	1-Way ANOVA	0.511	0.920, ^b^ 0.999, ^c^ 0.694 ^d^
Body mass index, mean (SD) e	21.06(3.48)	20.85(3.70)	21.03(3.42)	21.20(3.62)	1-Way ANOVA	0.099	0.792, ^b^ 0.315, ^c^ 0.164 ^d^

*SD* Standard deviation, *ANOVA* Analysis of variance, *MMSE* Mini-Mental State Examination, *NA* Not applicable

^a^ The Šídák multiple comparison test was used for all post hoc analyses

^b^ Increasing and long sleep duration vs. Stable adequate sleep duration

^c^ Increasing and long sleep duration vs. Decreasing and short sleep duration

^d^ Stable adequate sleep duration vs. Decreasing and short sleep duration

^e^ Calculated as weight in kilograms divided by height in meters squared

### Associations between sleep duration, sleep quality, food consumption, and cognitive function during follow-up

We assessed the longitudinal associations between sleep duration, sleep quality, food consumption, and global cognition scores (see Additional File 2, Table 2.4). After adjusting the confounding variables, our study did not detect a direct association between distinct trajectories of sleep quality or sleep duration and cognitive function. Furthermore, compared to stable moderate fruit and vegetable consumption, stable high consumption of fruit and vegetable was associated with enhanced cognitive function in older adults (0.088, 95%CI: 0.049 to 0.127), whereas decreasing low fruit and vegetable consumption was associated with detrimental effects on cognitive function (-0.141, 95%CI: -0.268 to -0.014). Compared to a slow decrease meat, fish, egg, and bean consumption, a rapid decrease in the consumption of meat, fish, egg, and bean was associated with lower cognitive function in older adults (-0.112, 95%CI: -0.155 to -0.069). Increasing milk and nuts consumption was associated with improved cognitive function in older adults (increasing milk consumption: 0.044, 95%CI: 0.011 to 0.087; increasing nuts consumption: 0.082, 95%CI: 0.037 to 0.127).

### Food consumption as a mediator in the relationship between sleep duration and cognitive function

**Fig. 2:**
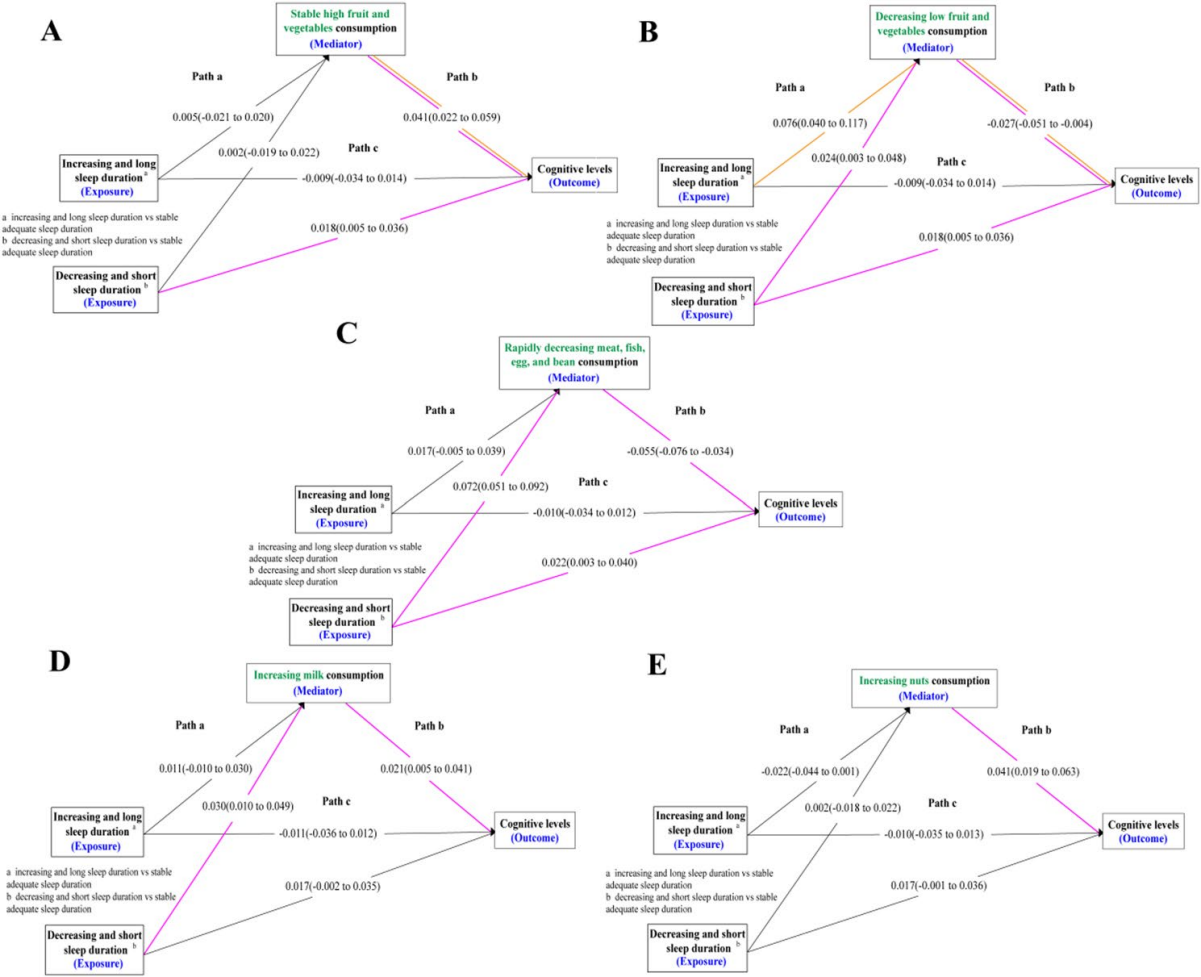
The Mediating Role of Food Consumption in Sleep Duration and Cognitive Function *Note* The results in the figure were presented as estimates and 95% confidence intervals. **A**, **B**, Fruit and vegetable consumption as a mediator in the association between increasing and long, decreasing and short sleep duration, and global cognitive scores using stable adequate sleep duration as the reference group controlled by covariates. **C**, Meat, fish, egg, and bean consumption as a mediator in the association between increasing increasing and long, decreasing and short sleep duration, and global cognitive scores using stable adequate sleep duration as the reference group controlled by covariates. **D**, Milk consumption as a mediator in the association between increasing and long, decreasing and short sleep duration, and global cognitive scores using stable adequate sleep duration as the reference group controlled by covariates. **E**, Nuts consumption as a mediator in the association between increasing and long, decreasing and short sleep duration, and global cognitive scores using stable adequate sleep duration as the reference group controlled by covariates

After controlling for confounders, we assessed food consumption in the relationship between sleep duration and cognitive function in older adults (Fig. 2). Compared to stable moderate fruit and vegetable consumption, our study found no significant mediating role of stable high fruit and vegetable consumption between sleep duration and cognitive levels, but decreasing low fruit and vegetable consumption did play a mediating role between sleep duration and cognitive levels. Both increasing and decreasing sleep duration led to a reduction in fruit and vegetable consumption, thereby lowering the cognitive levels of older adults. Notably, decreasing and short sleep duration (as low as 5 h per night) exhibited a direct positive association with cognitive function. Additionally, compared to a slow decline in the consumption of meat, fish, egg, and bean, a rapid decline meat, fish, egg, and bean consumption played a mediating role between decreasing and short sleep duration and cognitive levels. The shorter the sleep duration, the less intake of meat, fish, egg, and bean, which in turn lowered the cognitive levels. An increase in milk consumption played a complete mediating role between decreasing and short sleep duration and cognitive levels, meaning that a decrease in sleep duration increased milk consumption, thereby promoting the cognitive levels of older adults. No mediating effect was observed for nuts consumption between sleep duration and cognitive function (see Additional File 2, Table 2.5–2.8).

### Food consumption as a mediator in the relationship between sleep quality and cognitive function

**Fig. 3:**
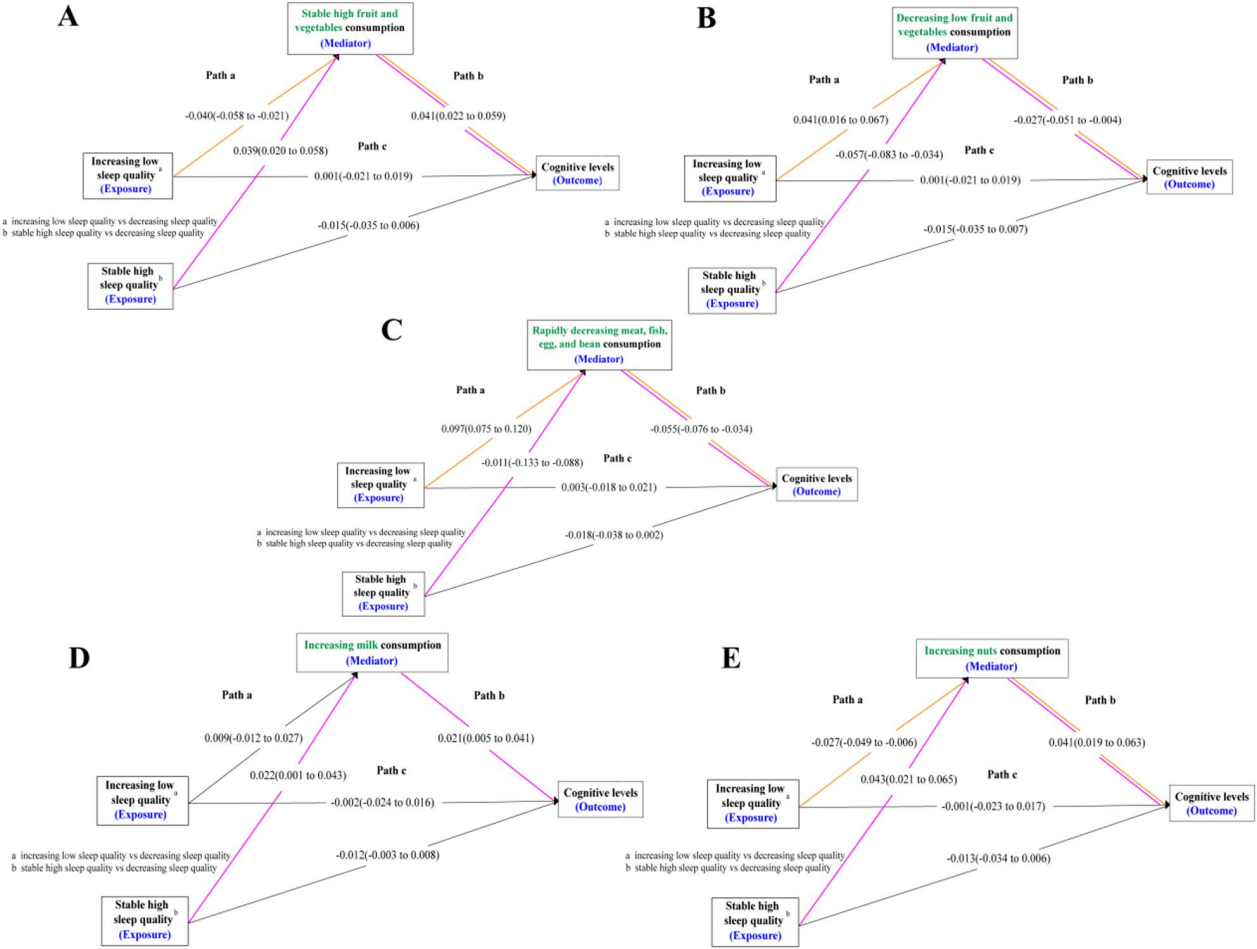
The Mediating Role of Food Consumption in Sleep Quality and Cognitive Function *Note* The results in the figure are presented as estimates and 95% confidence intervals. **A**, **B**, Fruit and vegetable consumption as a mediator in the association between increasing low, stable high sleep quality, and global cognitive scores using decreasing sleep quality as the reference group controlled by covariates. **C**, Meat, fish, egg, and bean consumption as a mediator in the association between increasing low, stable high sleep quality, and global cognitive scores using decreasing sleep quality as the reference group controlled by covariates. **D**, Milk consumption as a mediator in the association between increasing low,, stable high sleep quality, and global cognitive scores using decreasing sleep quality as the reference group controlled by covariates. **E**, Nuts consumption as a mediator in the association between increasing low, stable high sleep quality, and global cognitive scores using decreasing sleep quality as the reference group controlled by covariates

Similarly, food consumption played a mediating role in the relationship between sleep quality and cognitive function in older adults (Fig. 3). Compared to stable moderate fruit and vegetable consumption, both stable high and decreasing low fruit and vegetable consumption mediated the association between sleep quality and cognitive function. An increase in sleep quality, but with a relatively low overall score, led to a decrease in fruit and vegetable consumption, thereby lowering cognitive levels. Stable high sleep quality increased fruit and vegetable consumption, thereby increasing the cognitive levels of older adults. Compared to a slow decline in the intake of meat, fish, egg, and bean, a rapid decline meat, fish, egg, and bean consumption played a mediating role between sleep quality and cognitive level. Similarly, stable high sleep quality was associated with a slower decline in the intake of meat, fish, egg, and bean. Furthermore, a decline in the intake of meat, fish, egg, and bean led to a decrease in the cognitive level. Compared to not drinking milk, stable high sleep quality increased milk consumption, thereby improving cognitive levels. Similarly, compared to not eating nuts, stable high sleep quality increased nuts consumption, thereby improving cognitive levels (see Additional File 2, Table 2.5–2.8).

## Discussion

By examining longitudinal trajectories, our study provided a more nuanced understanding of how sleep and food consumption patterns influenced cognitive function in older adults. The key findings indicated that the trajectories of these behaviors was predictive of cognitive outcomes. And we examined the effect of food consumption on the relationships between sleep duration, sleep quality, and cognitive function among adults aged 65 years and above. Both increasing and decreasing sleep duration led to a reduction in fruit and vegetable consumption, thereby lowering the cognitive levels of older adults. Additionally, the shorter the sleep duration, the less intake of meat, fish, egg, and bean, which in turn lowered the cognitive levels. However, a decrease in sleep duration increased milk consumption, thereby promoting the cognitive levels of older adults. Stable high sleep quality increased fruit, vegetable, meat, fish, egg, bean, milk, and nuts consumption, thereby increasing the cognitive levels of older adults.

Some previous research showed that sleep duration, sleep quality, and dietary patterns were related. To our knowledge, a study that examined the effects of the Energy-density Dietary Inflammatory Index (E-DII™) on objectively and subjectively measured sleep among police officers showed that more pro-inflammatory diets were associated with higher wake-after-sleep-onset [40]. Some studies suggested that sleep timing and quality influenced dietary choices [41–43]. In adolescents, the odds of missing breakfast were significantly higher in children who reported poor sleep or later bedtimes, while the odds of junk food consumption were significantly higher in children reporting later weeknight bedtimes [44]. In a cohort of Italian adults, individuals in the highest quartile of the dietary inflammatory index were less likely to have adequate sleep quality [41].

In addition, diet plays a crucial role in cognitive function. Some studies investigated dietary patterns associated with cognitive functions in older Asian adults, including China [45], Korean [46], and Japanese [47] populations in a cross-sectional and longitudinal design. For instance, older adults with high intakes of vegetable and fruits, soy products, and legumes, had a reduced risk of cognitive impairment in a cross-sectional study of China [45]. Similarly, higher intakes of vegetable and their constituent nutrients were associated with a lower risk of dementia in Japanese older adults [47]. These findings also followed previous research conducted in Western countries. In a French older population, participants with the highest consumption of fruit, whole grains, fresh dairy products, and vegetable, demonstrated better cognitive functioning [48]. The older adults with higher intakes of salad dressing, nuts, fish, tomatoes, poultry, fruits, dark and green leafy vegetable, and a lower intake of high-fat dairy products, red meat, and butter, had a reduced risk of Alzheimer’s disease in a cohort study of U.S [49].

In a community–based setting, it showed that in long sleepers (> 8 h), cognitive impairment was significantly associated with low cystine, proline, and serine intake [50]. However, there were fewer studies on the role of food consumption in the correlation between sleep and cognitive performance. Our findings supported the mediating effect of food consumption on the relationships between sleep duration, sleep quality, and cognitive levels in the old-aged Chinese population. Both increasing and decreasing sleep duration led to reduced fruit and vegetable consumption, negatively impacting cognition. A rapid decline in meat, fish, egg, and bean consumption also mediated the negative effect of reduced sleep duration on cognition. Conversely, increased milk consumption acted as a complete mediator, improving cognition despite shorter sleep. Similar mediation effects were observed for sleep quality. Both high and increasing fruit and vegetable consumption mediated the link between sleep quality and cognition. However, improved sleep quality with low overall scores paradoxically decreased fruit and vegetable intake, lowering cognition, while stable, high-quality sleep increased fruit and vegetable intake, boosting cognition. A rapid decline in meat, fish, egg, and bean consumption mediated the negative effect of sleep quality on cognition. Stable, high-quality sleep also increased milk and nuts consumption, both positively impacting cognition.

Traditional Chinese diets traditionally emphasize vegetables, aligning with the cognitive benefits observed in individuals with stable, high-quality sleep, which likely reinforces adherence to healthy dietary patterns. However, modern pressures like busy lifestyles, a growing reliance on convenience foods, and the time required for fruit and vegetable preparation, are plausible explanations for the reduced intake of these items often associated with poor sleep, whether due to low sleep quality or altered sleep duration. Meat, fish, egg, and bean sever as fundamental sources of high-quality protein, iron, and B vitamins within Chinese dietary patterns. Stable high sleep quality likely supports the physiological capacity and socioeconomic stability needed to maintain adequate intake of these nutrient-dense traditional staples. Although traditional Chinese diets historically featured lower milk consumption, intake is increasing, particularly among older adults. The finding that milk intake increases with decreased sleep duration and benefits cognition suggests it may act as a compensatory nutritional strategy. Individuals experiencing sleep loss might consciously increase milk consumption, possibly perceiving its calming effects (due to tryptophan) or its comprehensive nutritional profile (often fortified with Vitamin D and calcium). Stable good sleep facilitates the consistent adoption of this increasingly promoted dietary component. Similarly, nuts are recognized in Chinese food therapy and modern nutrition for their health benefits (unsaturated fats, antioxidants). Nuts intake, often as snacks or in modest quantities, appears less susceptible to fluctuations in sleep duration. However, the link between stable high sleep quality and increased nuts consumption suggests that sustained well-being fosters dietary patterns incorporating perceived “health foods”.

Generally speaking, our findings confirmed that food consumption played a significant mediating role in the relationships between both sleep duration and sleep quality with cognitive function among older adults. Crucially, stable high-quality sleep promoted dietary patterns conducive to positive cognitive outcomes. This includes supporting adherence to traditional vegetable-centric diets and facilitating the consumption of nutritionally beneficial foods increasingly promoted in China, such as meat, fish, egg, bean, milk, and nuts.

### Limitations

This study has several limitations. First, sleep parameters in the CLHLS database were self-reported, which may introduce information bias and affect study results compared to objective measurements. Second, we conducted the study by excluding data due to missing data or being lost to follow-up or ineligible in the present study, which may have resulted in selection bias. Third, we used the MMSE to assess cognitive levels in older adults, which was less reliable than clinical diagnostic results. Although the results of these tests cannot replace the diagnosis based on clinical examination, they had been used to study the relationships between cognitive function and various risk factors [21]. And the cognitive levels were not categorized by different degrees of severity. The reason is that the CLHLS did not include the Montreal Cognitive Assessment (MoCA) scale, which precluded the assessment of mild cognitive impairment. Additionally, our study did not conduct a specific analysis of the functional aspects of cognitive levels. The CLHLS used one question to assess sleep quality, and there was no clearly defined time frame for the evaluation, which might lead to an underestimation of sleep disorders. Fourth, the use of GBTM, while advantageous for identifying distinct longitudinal patterns, had inherent limitations. Although the model fit was primarily determined by BIC and AIC, and most groups showed acceptable entropy values (e.g., 0.815 for sleep quality), we acknowledged that the relatively lower entropy values for some trajectories (e.g., 0.401 for the two-trajectory sleep duration model) indicated a degree of uncertainty in individual group assignment. This suggested a potential for misclassification bias, where some participants might not perfectly align with their assigned trajectory. However, it was important to note that the primary goal of GBTM was to summarize population-level trends rather than to achieve perfect prediction for every individual. And it was important to consider the interrelationship between sleep duration and sleep quality. Although our statistical models adjusted for both parameters simultaneously to estimate their independent effects, we could not rule out residual confounding. Finally, there may be residual confounding factors that we had not controlled for, including sleep apnea syndromes, sleep medications, and anxiety, because these variables were not included in the 2005–2014 CLHLS data. Future studies should consider these limitations and attempt to address them to provide a more comprehensive understanding of this complex relationship.

## Conclusions

Our study focused on the role of food consumption in causing cognitive decline due to unhealthy sleep patterns among older adults, there was evidence to suggest that food consumption mediated the relationships between sleep duration, sleep quality, and cognitive function. Highlighting the impact of food consumption in the associations of sleep duration and quality on cognitive function in older adults is necessary to understand the contribution of healthy sleep to public health. By identifying modifiable potential factors, it is helpful to carry out interventions aimed at enhancing the sleep quality of older adults, which may then delay the alterations in their cognitive function. Overall, our study provides new insights into improving cognitive levels in older adults by co-managing their sleep and dietary patterns.

## Supplementary Information

Supplementary Material 1.

Supplementary Material 2.

## Data Availability

The data that support the findings of this study are available from the Chinese Longitudinal Healthy Longevity Survey (CLHLS) by the Center for Healthy Aging and Development, Peking University. https://doi.org/10.18170/DVN/WBO7LK.

## Abbreviations

CLHLS: Chinese Longitudinal Healthy Longevity Survey
MMSE: Mini-Mental State Examination
SD: Standard Deviation
CBT: Cognitive Behavioural Therapy
BMI: Body Mass Index
