# Supplementary material for: What is the role of food consumption in the relationships between sleep duration, sleep quality, and cognitive function? A study among Chinese older adults
Source: BMC Geriatr. 2026 Feb 27;26:453. doi: 10.1186/s12877-026-07037-1 (PMC13041492; doi:10.1186/s12877-026-07037-1)
Supplement: Supplementary file 1 — Supplementary Material 1. [file 12877_2026_7037_MOESM1_ESM.docx]

**Additional File 1 - Supplementary methods**

1. **Cognitive assessment**

Cognitive assessment was conducted in face-to-face interviews and covered five domains, orientation, registration, attention and calculation, recall, and language.

**1.1 Orientation**

This section consisted primarily of five questions: “What time of day is it right now (morning, afternoon, evening)?” “What is the month (Western or Chinese calendar) right now?” “What is the date (Chinese calendar day and month) of the mid-autumn festival?” “What is the season right now, spring, summer, fall, winter?” “What is the name of this district or town?” One point for each correct answer, zero points for each incorrect answer.

**1.2 Registration**

The interviewers said three items: table, apple, clothes, and the interviewee repeated them in order. If answers were insufficient or incorrect on the first attempt, repeated the names of all objects until the interviewee was able to name all three of them (6 attempts at maximum).

**1.3 Attention and calculation**

The interviewers asked the interviewee to spend 3 dollars from 20 dollars, then individuals must spend 3 dollars from the number they arrived at and continue to spend 3 dollars until individuals were asked to stop. Besides, the interviewee was asked to draw the figure on B Card. If all the sides and angles were correct and the figure in the middle was a quadrangle, it counts as one point; otherwise, as zero points.

**1.4 Recall**

The interviewees were asked by the interviewer to remember and describe the three items mentioned earlier (table, apple, and clothes). Just state the correct names, regardless of the order.

**1.5 Language**

Giving the interviewees a pen and a watch, then asking what these objects were called. In addition, the interviewees were asked to repeat the following sentence: “What you plant, what you will get”. The interviewers gave the interviewee a piece of paper, then the individuals needed to take the paper using the right hand, fold it in the middle using both hands, and place the paper on the floor.

1. **Covariates**

Additional variables included as potential confounders in these analyses were collected in face-to-face interviews.

**2.1 Sociodemographic information**

1. Sex: female, male.
2. Birth date (for age calculation).
3. District: Eastern China, Central China, Western China. Central China: North China (Beijing, Tianjin, Hebei, Shanxi); Northeast China (Liaoning, Jilin, Heilongjiang); Central China (Henan, Hubei, Hunan). Eastern China: East China (Shanghai, Jiangsu, Zhejiang, Anhui, Fujian, Jiangxi, Shandong); South China (Guangdong, Guangxi). Western China: Southwest China (Chongqing, Sichuan); Northwest China (Shanxi).
4. Residency type: city, town, rural.
5. Main occupation before the age of 60: non-manual workers (professional and technical personnel; governmental, institutional or managerial personnel; military personnel) , manual workers (commerical, serviceor or industrial worker; agriculture, forestry, animal husbandry of fishery worker), others (self-employer; houseworker).
6. Current marriage status: married; separated; divorced; widowed; never married.

**2.2 Health-related behavior**

(1) We categorized individuals’ smoking, drinking and exercise habits into three categories based on their past and current behavior: present (always smoked/ drank/ exercised from past to present, never smoked/drank/exercised in the past but do so now); previous (smoked/drank/ exercised in the past but not now); and none (never smoked/drank/ exercised from past to present).

(2) The frequency of participation in social activities, categorized into two levels (yes and no), was included.

**2.3 Health status**

(1) Chronic diseases: In the survey, respondents were asked whether they had suffered from stroke/cerebrovascular, diabetes, hypertension, heart attack, tuberculosis, arthritis, cataract, glaucoma, cancer, Parkinson’s disease, etc. The number of chronic diseases for each respondent was categorized as 0, 1, and ≥2.

(2) Systolic and diastolic blood pressure (the interviewers measured the blood pressure of the elderly twice, with at least 1 minute between the two measurements).

(3) Body mass index was calculated from the weight (kilograms) and height (centimeters) of the individuals using the following formula: weight (kg) / height^2^ (m^2^).

(4) In addition, heart rate (beats/min) was measured.

(5) Depression: Depressive symptoms in CLHLS were assessed by a five-item scale. Information about depressive symptoms was obtained by asking respondents the following questions: “1. Do you always see the bright side of things? 2. Are you as happy as you were at an early age? 3. Do you often feel lonely or isolated? 4. Do you feel the older you get, the less useful you are? 5. Do you often feel afraid or anxious?” These five questions were frequently used by previous studies to identify depressive symptoms[1–3]. The first two questions measured positive feelings, while the other three questions measured negative feelings. Response options included always, often, sometimes, seldom, and never. The score for each response ranged from 0 to 4. The two positive questions were added to calculate the total score, and the three negative questions were reversely coded. Accordingly, the total score of five items ranged from 0 to 20, and a higher score indicated more depressive symptoms.

**References**

1. Ren Z, Wang S, He M, et al. The effects of living arrangements and leisure activities on depressive symptoms of Chinese older adults: Evidence from panel data analysis. *J Affect Disord*. 2024;349:226-233. doi:10.1016/j.jad.2024.01.077

2. Shen K, Zhang B, Feng Q. Association between tea consumption and depressive symptom among Chinese older adults. *BMC Geriatr*. 2019;19:246. doi:10.1186/S12877-019-1259-Z,

3. Guo H, Zhang Y, Wang Z, Shen H. Sleep Quality Partially Mediate the Relationship Between Depressive Symptoms and Cognitive Function in Older Chinese: A Longitudinal Study Across 10 Years. *Psychol Res Behav Manag*. 2022;15:785-799. doi:10.2147/PRBM.S353987
