# Supplementary material for: What is the role of food consumption in the relationships between sleep duration, sleep quality, and cognitive function? A study among Chinese older adults
Source: BMC Geriatr. 2026 Feb 27;26:453. doi: 10.1186/s12877-026-07037-1 (PMC13041492; doi:10.1186/s12877-026-07037-1)
Supplement: Supplementary file 2 — Supplementary Material 2. [file 12877_2026_7037_MOESM2_ESM.docx]

**Additional File 2**

**Figure 2.1.** Sleep duration trajectories in older adults completing at least three measurements


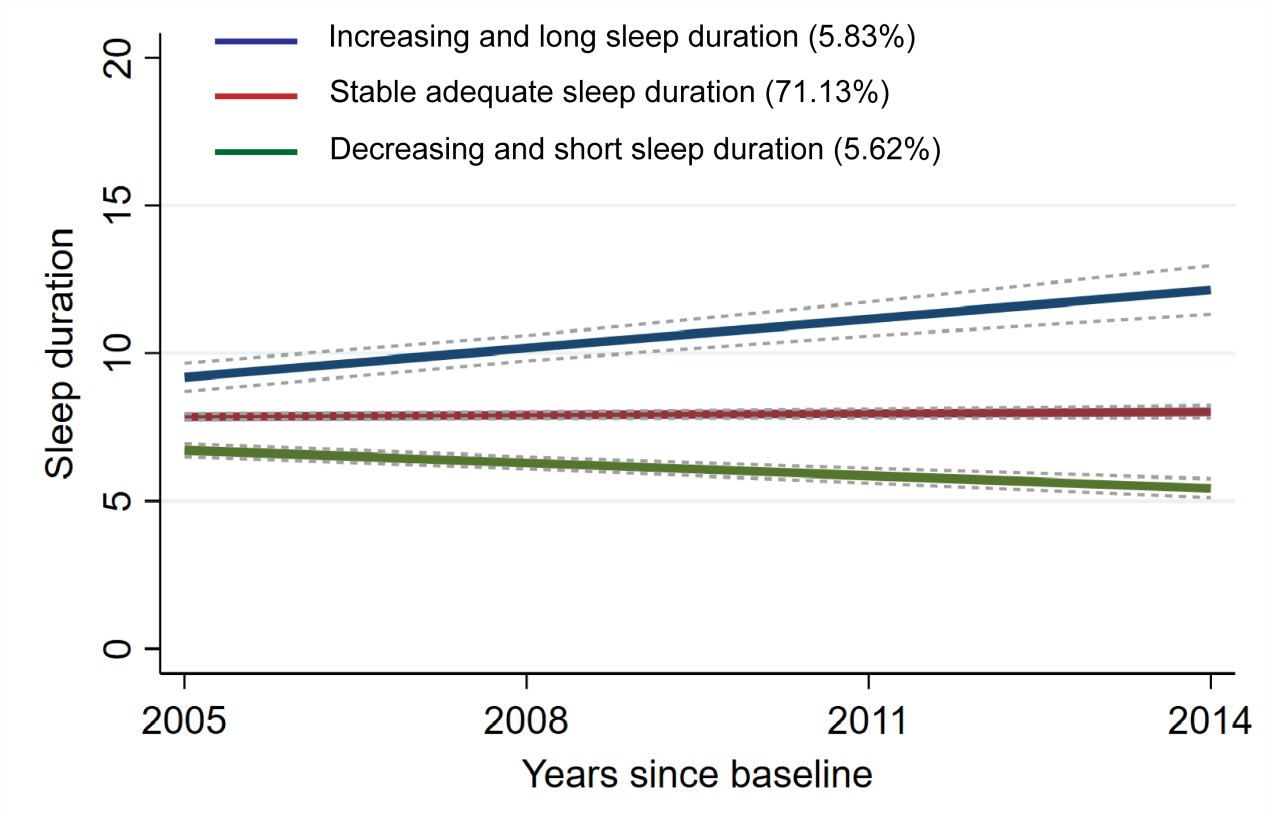


**Figure 2.2.** Sleep quality trajectories in older adults completing at least three measurements


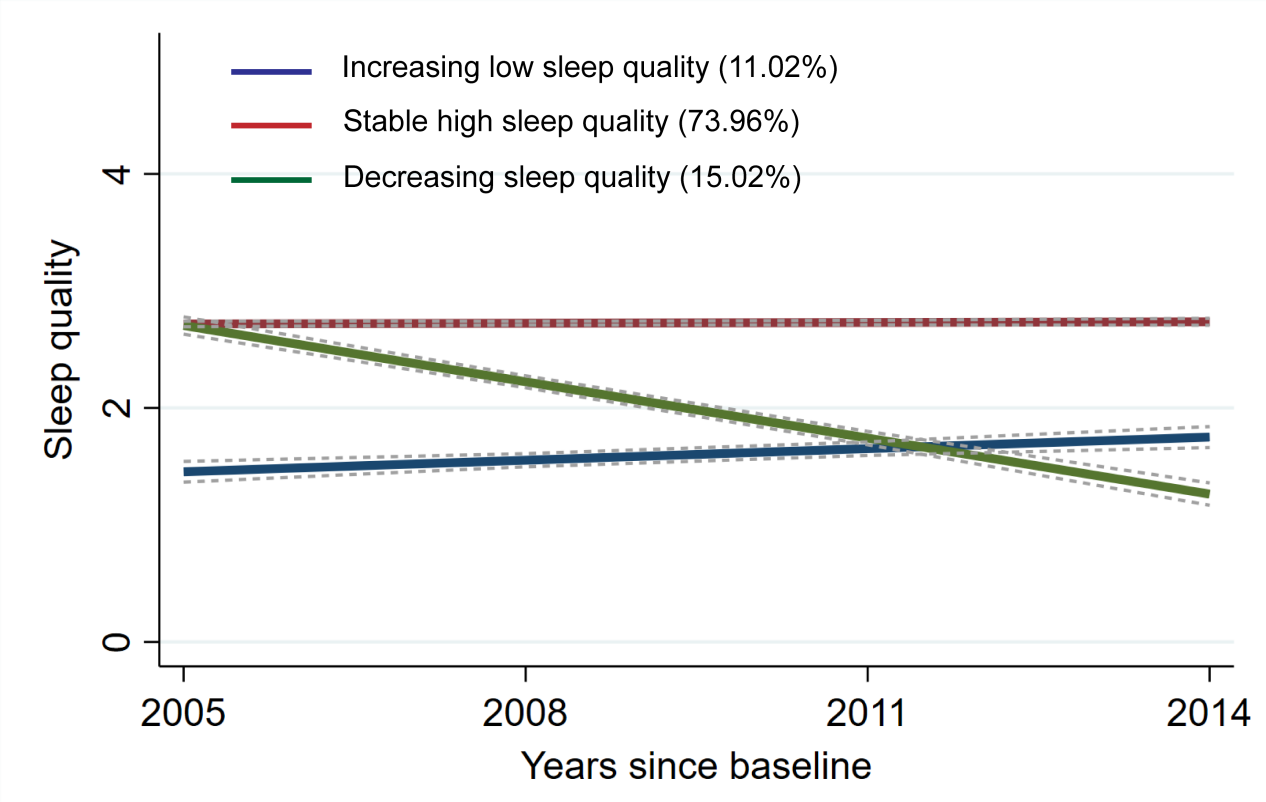


**Figure 2.3.** Fruit and vegetable consumption trajectories in older adults completing at least three measurements


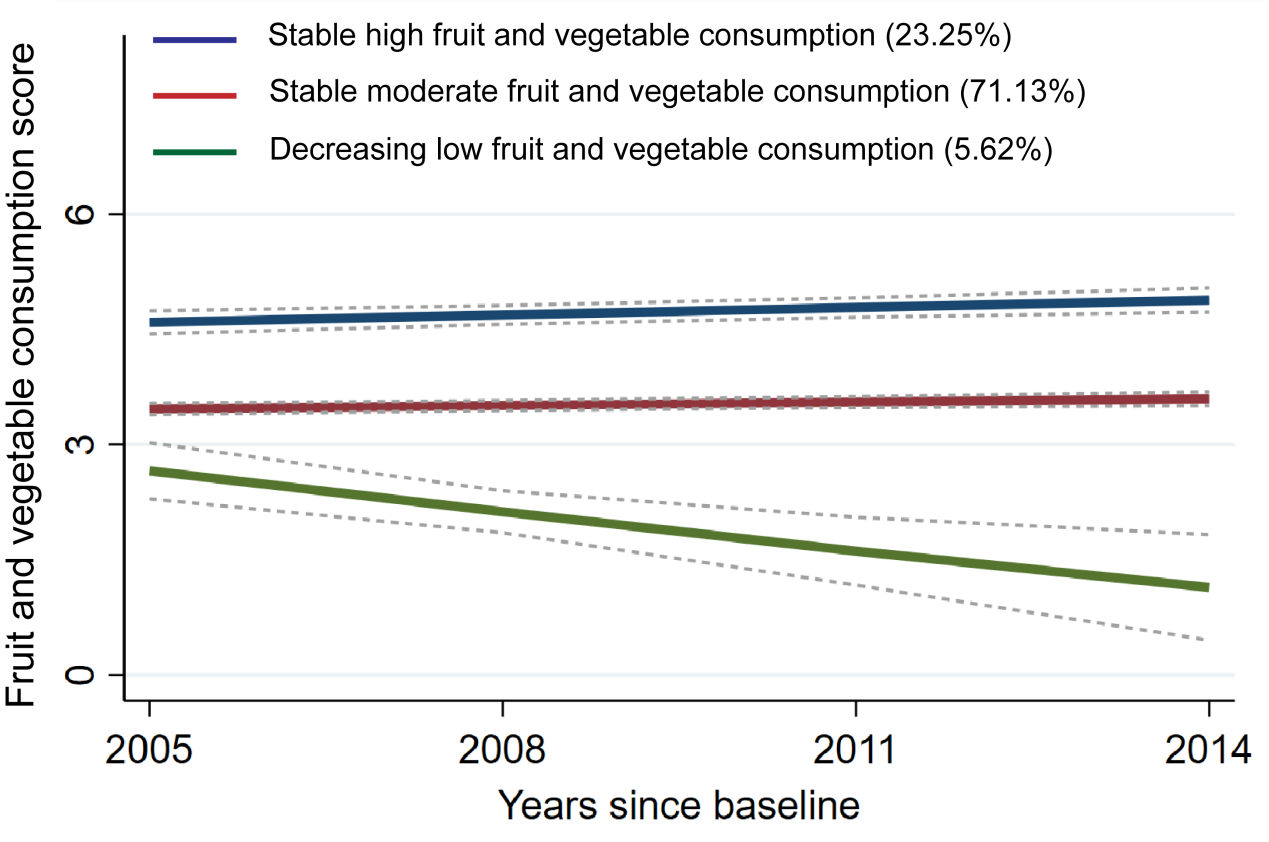


**Figure 2.4.** Meat, fish ,egg, and bean consumption trajectories in older adults completing at least three measurements


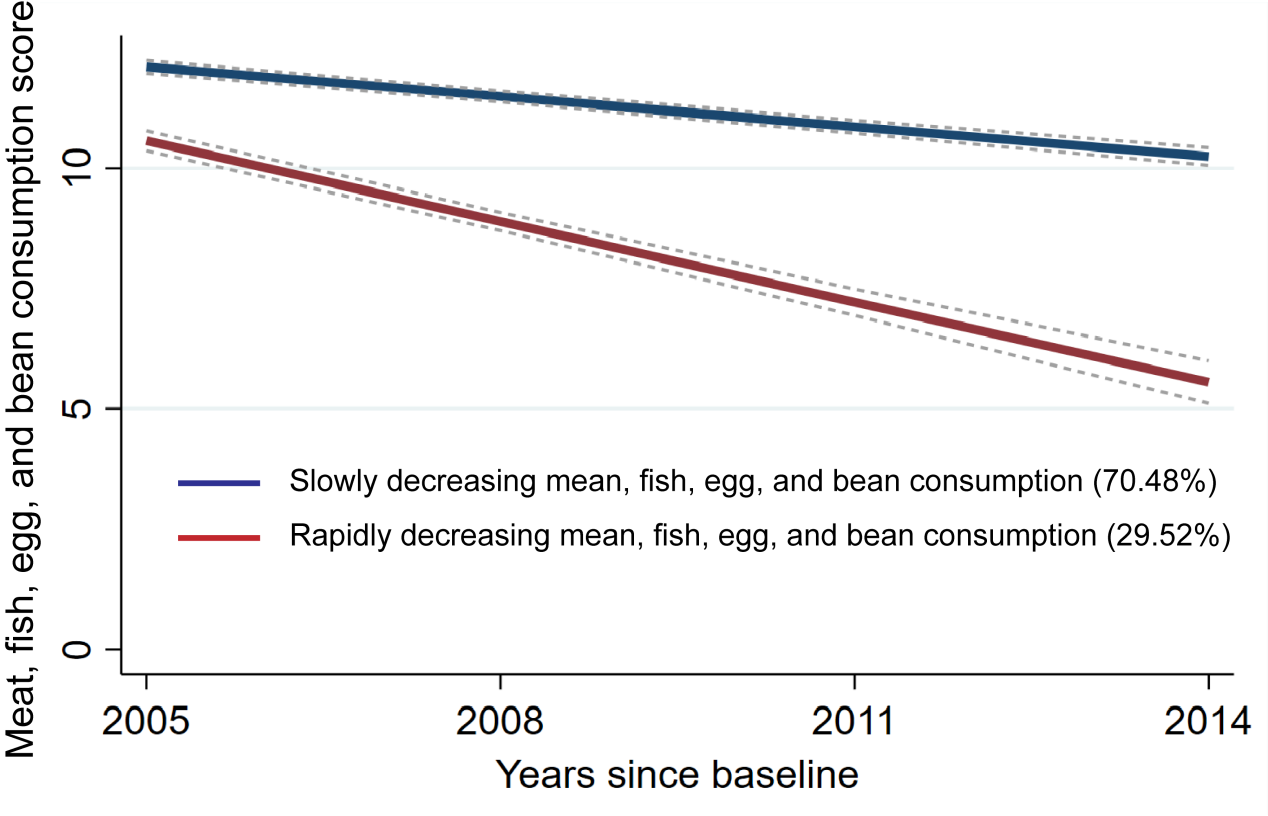


**Figure 2.5.** Milk consumption trajectories in older adults completing at least three measurements


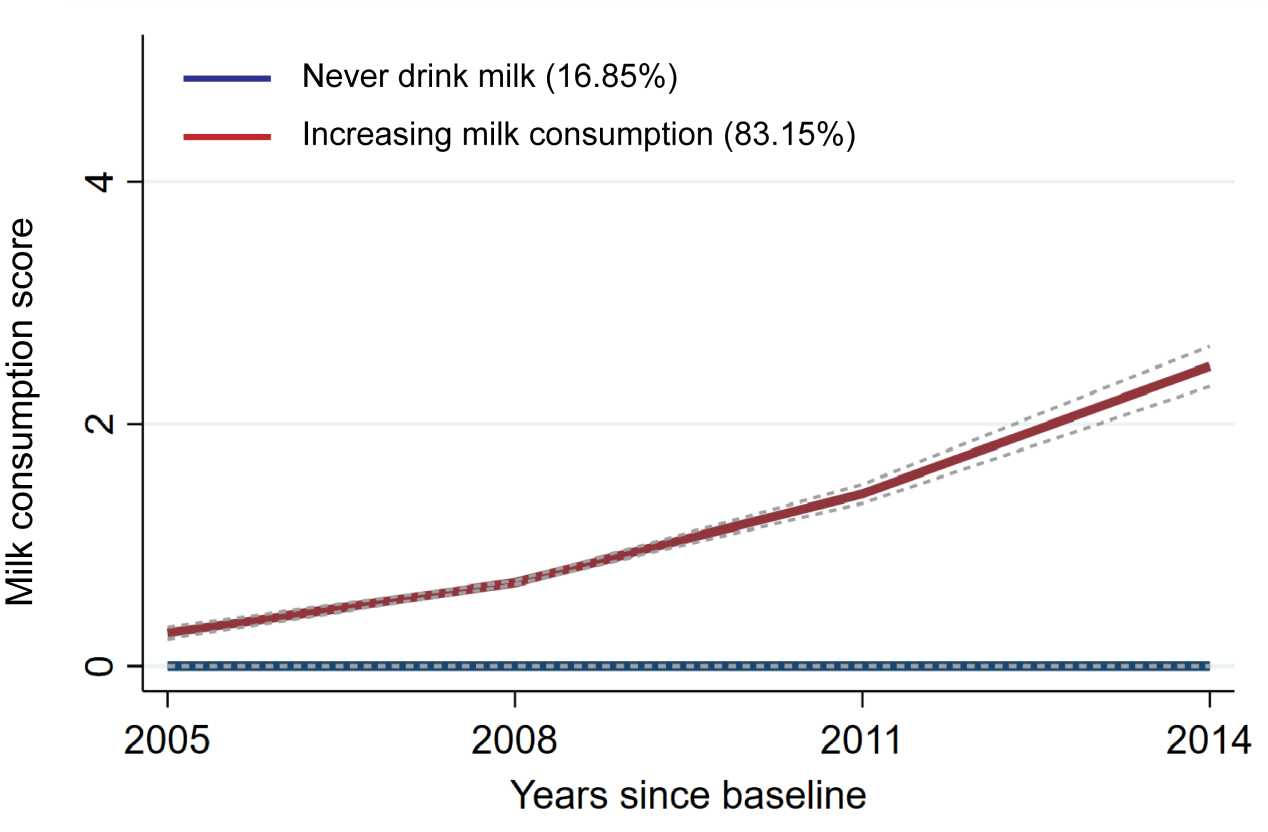


**Figure 2.6.** Nuts consumption trajectories in older adults completing at least three measurements


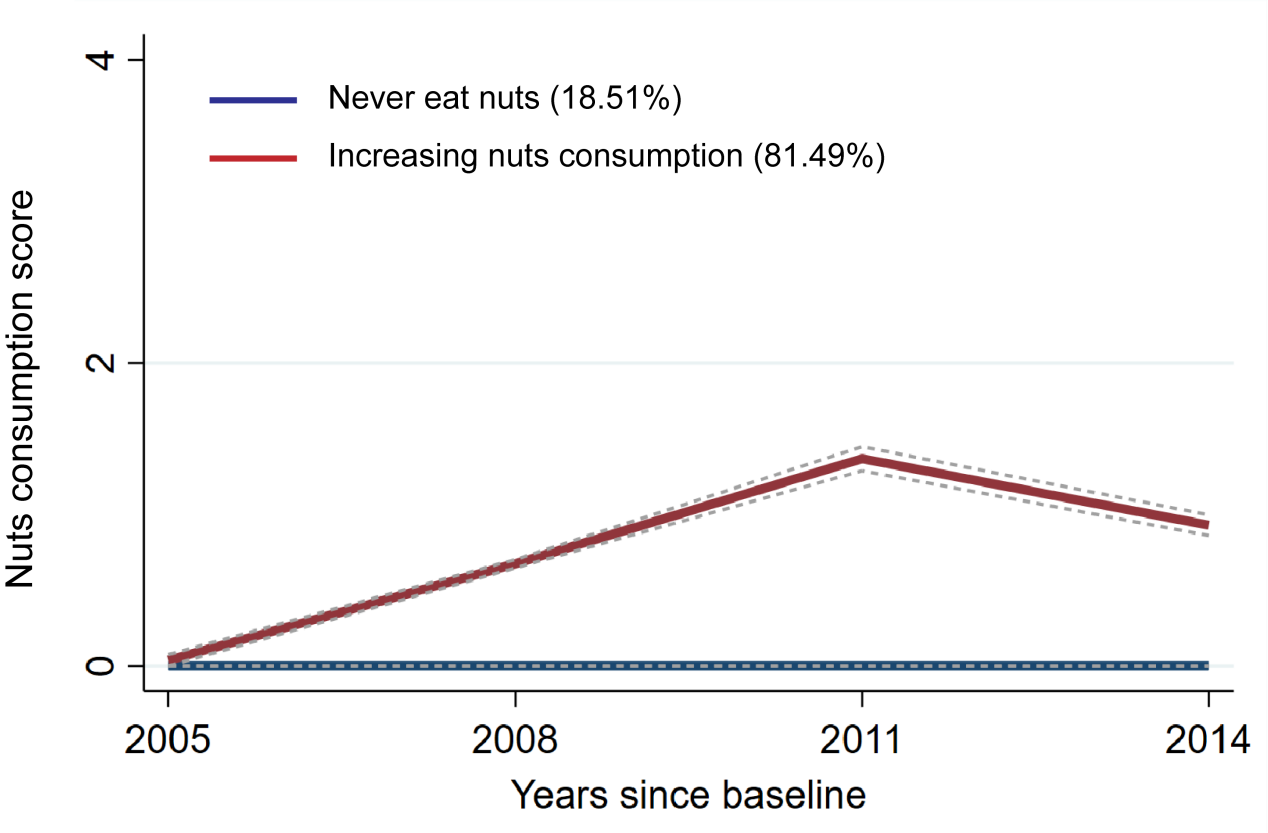


**Table 2.1.** Estimated parameters for sleep parameters and food consumption by group-based trajectory model

| **Outcomes** | **Orders** | **BIC** | **AIC** | **ll** | **Entropy** | **PPGM1(%)** | **PPGM2(%)** | **PPGM3(%)** | **PPGM4(%)** |
| --- | --- | --- | --- | --- | --- | --- | --- | --- | --- |
| **Sleep duration** |  |  |  |  |  |  |  |  |  |
| Three trajectories | 1 1 1 | -18867.23 | -18841.12 | -18832.12 | 0.639 | 5.83 | 68.56 | 25.61 |  |
| Two trajectories | 1 1 | -18935.15 | -18917.75 | -18911.75 | 0.401 | 48.53 | 51.47 |  |  |
| One trajectories | 1 | -19144.43 | -19135.73 | -19132.73 |  | 100.00 |  |  |  |
| **Sleep Quality** |  |  |  |  |  |  |  |  |  |
| Three trajectories | 1 1 1 | -8550.94 | -8524.83 | -8515.83 | 0.815 | 11.02 | 73.96 | 15.02 |  |
| Two trajectories | 1 1 | -8706.43 | -8689.02 | -8683.02 | 0.809 | 20.95 | 79.05 |  |  |
| One trajectories | 1 | -9359.04 | -9350.33 | -9347.33 |  | 100.00 |  |  |  |
| **Fruit and vegetable consumption** |  |  |  |  |  |  |  |  |  |
| Four trajectories | 1 1 1 1 | -14488.98 | -14454.17 | -14442.17 | 0.738 | 21.84 | 3.16 | 1.66 | 73.34 |
| Three trajectories | 1 1 1 | -14495.62 | -14469.50 | -14460.50 | 0.785 | 23.25 | 71.13 | 5.62 |  |
| Two trajectories | 1 1 | -14557.82 | -14540.42 | -14534.42 | 0.455 | 64.17 | 35.83 |  |  |
| One trajectories | 1 | -14751.34 | -14742.64 | -14739.64 |  | 100.00 |  |  |  |
| **Meat, fish, egg, and bean consumption** |  |  |  |  |  |  |  |  |  |
| Two trajectories | 1 1 | -21964.60 | -21947.19 | -21941.19 | 0.670 | 70.48 | 29.52 |  |  |
| One trajectories | 1 | -22238.37 | -22229.66 | -22226.66 |  | 100.00 |  |  |  |
| **Milk consumption** |  |  |  |  |  |  |  |  |  |
| Two trajectories | 2 2 | -10047.85 | -10024.64 | -10016.64 | 0.701 | 25.95 | 74.05 |  |  |
| Two trajectories | 1 1 | -10605.06 | -10587.65 | -10581.65 | 0.735 | 16.85 | 83.15 |  |  |
| One trajectories | 1 | -10752.79 | -10744.09 | -10741.09 |  | 100.00 |  |  |  |
| **Nuts consumption** |  |  |  |  |  |  |  |  |  |
| Two trajectories | 2 2 | -8849.96 | -8826.75 | -8818.75 | 0.611 | 8.11 | 91.89 |  |  |
| Two trajectories | 1 2 | -8799.49 | -8779.18 | -8772.18 | 0.721 | 18.51 | 81.49 |  |  |
| One trajectories | 1 | -9265.36 | -9256.66 | -9253.66 |  | 100.00 |  |  |  |

*Note* BIC = Bayesian information criterion; AIC: Akaike’ s Information Criterion; ll: log-likelihood; PPGM1: Predicted Probability of Group Membership in Group 1; PPGM2: Predicted Probability of Group Membership in Group 2; PPGM3: Predicted Probability of Group Membership in Group 3; PPGM4: Predicted Probability of Group Membership in Group 4

**Table 2.2.** Baseline Characteristics of Participants in the Study (Stratified by Sleep Quality)

| **Characteristic** | **Total (*n*=8724)** | **Participants, No. (%)** | | | | | |
| --- | --- | --- | --- | --- | --- | --- | --- |
|  |  | **Sleep quality trajectories** | | | | | |
|  |  | **Increasing low sleep quality (*n*=980)** | **Stable high sleep quality (*n*=6661)** | **Decreasing sleep quality (*n*=1083)** | **Statistical test** | ***p-*value** | ***p-*value for post hoc test** ^a^ |
| Age, mean (SD), y | 79.31(8.66) | 77.81(7.32) | 79.55(8.89) | 79.18(8.20) | 1-Way ANOVA | <0.001 | <0.001, ^b, c^ 0.489 ^d^ |
| Sex |  |  |  |  |  |  |  |
| Female | 4334(49.68) | 318(32.45) | 3563(53.49) | 453(41.83) | χ^2^ | <0.001 | NA |
| Male | 4390(50.32) | 662(67.55) | 3098(46.51) | 630(58.17) |  |  |  |
| District |  |  |  |  |  |  |  |
| Eastern China | 4984(57.13) | 538(54.90) | 3846(57.74) | 600(55.40) | χ^2^ | 0.270 | NA |
| Central China | 2253(25.83) | 258(26.32) | 1700(25.52) | 295(27.24) |  |  |  |
| Western China | 1487(17.04) | 184(18.78) | 1115(16.74) | 188(17.36) |  |  |  |
| Marital status |  |  |  |  |  |  |  |
| Married | 4483(51.39) | 475(48.47) | 3474(52.15) | 534(49.31) | χ^2^ | 0.009 | NA |
| Divorced | 32(0.37) | 5(0.51) | 22(0.34) | 5(0.46) |  |  |  |
| Widowed | 4135(47.4) | 485(49.49) | 3109(46.67) | 541(49.95) |  |  |  |
| Never married | 74(0.85) | 15(1.53) | 56(0.84) | 3(0.28) |  |  |  |
| Residence type |  |  |  |  |  |  |  |
| City | 1425(16.33) | 157(16.02) | 1138(17.08) | 130(12.00) | χ^2^ | 0.001 | NA |
| Town | 2552(29.25) | 269(27.45) | 1945(29.20) | 338(31.21) |  |  |  |
| Rural | 4747(54.41) | 554(56.53) | 3578(53.72) | 615(56.79) |  |  |  |
| Main occupation before 60 y |  |  |  |  |  |  |  |
| Non-manual workers | 888(10.18) | 79(8.06) | 733(11.00) | 76(7.02) | χ^2^ | <0.001 | NA |
| Manual workers | 6992(80.15) | 808(82.45) | 5292(79.45) | 892(82.36) |  |  |  |
| Other | 844(9.67) | 93(9.49) | 636(9.55) | 115(10.62) |  |  |  |
| Smoking status |  |  |  |  |  |  |  |
| Present | 1967(22.55) | 182(18.57) | 1562(23.45) | 223(20.59) | χ^2^ | <0.001 | NA |
| Previous | 1529(17.53) | 121(12.35) | 1249(18.75) | 159(14.68) |  |  |  |
| None | 5228(59.93) | 677(69.08) | 3850(57.80) | 701(64.73) |  |  |  |
| Drinking status |  |  |  |  |  |  |  |
| Present | 1889(21.65) | 158(16.12) | 1543(23.16) | 188(17.35) | χ^2^ | <0.001 | NA |
| Previous | 1323(15.17) | 103(10.51) | 1072(16.10) | 148(13.67) |  |  |  |
| None | 5512(63.18) | 719(73.37) | 4046(60.74) | 747(68.98) |  |  |  |
| Exercising status |  |  |  |  |  |  |  |
| Present | 3489(39.99) | 366(37.35) | 2750(41.29) | 373(34.44) | χ^2^ | <0.001 | NA |
| Previous | 978(11.21) | 96(9.80) | 766(11.50) | 116(10.71) |  |  |  |
| None | 4257(48.8) | 518(52.85) | 3145(47.21) | 594(54.85) |  |  |  |
| Social activities |  |  |  |  |  |  |  |
| Yes | 1690(19.37) | 140(14.29) | 1375(20.64) | 175(16.16) | χ^2^ | <0.001 | NA |
| No | 7034(80.63) | 840(85.71) | 5286(79.36) | 908(83.84) |  |  |  |
| Fruit and vegetable consumption |  |  |  |  |  |  |  |
| Stable high fruit and vegetable consumption | 1871(21.45) | 150(15.31) | 1530(22.97) | 191(17.64) | χ^2^ | <0.001 | NA |
| Stable moderate fruit and vegetable consumption | 6611(75.78) | 784(80.00) | 4983(74.81) | 844(77.93) |  |  |  |
| Decreasing low fruit and vegetable consumption | 242(2.77) | 46(4.69) | 148(2.22) | 48(4.43) |  |  |  |
| Meat, fish, egg and bean consumption |  |  |  |  |  |  |  |
| Slowly decreasing | 6411(73.49) | 562(57.35) | 5146(77.26) | 703(64.91) | χ^2^ | <0.001 | NA |
| Rapidly decreasing | 2313(26.51) | 418(42.65) | 1515(22.74) | 380(35.09) |  |  |  |
| Milk consumption |  |  |  |  |  |  |  |
| Never drink milk | 2146(24.6) | 240(24.49) | 1583(23.77) | 323(29.82) | χ^2^ | 0.001 | NA |
| Increasing milk consumption | 6578(75.4) | 740(75.51) | 5078(76.23) | 760(70.18) |  |  |  |
| Nuts consumption |  |  |  |  |  |  |  |
| Never eat nuts | 2382(27.3) | 303(30.92) | 1732(26.00) | 347(32.04) | χ^2^ | <0.001 | NA |
| Increasing nuts consumption | 6342(72.7) | 677(69.08) | 4929(74.00) | 736(67.96) |  |  |  |
| Number of chronic diseases |  |  |  |  |  |  |  |
| 0 | 3733(42.79) | 340(34.69) | 2982(44.77) | 411(37.95) | χ^2^ | <0.001 | NA |
| 1 | 2360(27.05) | 279(28.47) | 1786(26.81) | 295(27.24) |  |  |  |
| ≥2 | 2631(30.16) | 361(36.84) | 1893(28.42) | 377(34.81) |  |  |  |
| Blood pressure, mean (SD), mm Hg |  |  |  |  |  |  |  |
| Systolic | 135.12(23.03) | 135.84(23.87) | 134.96(23.29) | 135.42(20.55) | 1-Way ANOVA | 0.480 | 0.599, ^b^ 0.966, ^c^ 0.905 ^d^ |
| Diastolic | 80.97(17.31) | 80.83(17.14) | 80.95(18.03) | 81.28(12.20) |  | 0.811 | 0.996, ^b^ 0.912, ^c^ 0.915 ^d^ |
| Global Cognitive scores, MMSE, mean (SD) | 20.32(3.24) | 20.02(3.27) | 20.39(3.21) | 20.10(3.37) | 1-Way ANOVA | <0.001 | 0.002, ^b^ 0.932, ^c^ 0.016 ^d^ |
| Depression scores, mean (SD) | 6.48(3.49) | 7.63(3.70) | 6.16(3.40) | 7.41(3.51) | 1-Way ANOVA | <0.001 | 0.386, ^c^ <0.001 ^b, d^ |
| Sleep duration per night, mean (SD), h | 7.58(2.17) | 5.97(2.26) | 7.95(1.99) | 6.75(2.24) |  |  |  |
| Heart rate, mean (SD), beats/min | 74.01(15.15) | 74.71(16.44) | 73.90(15.76) | 74.03(8.80) | 1-Way ANOVA | 0.294 | 0.313, ^b^ 0.667, ^c^ 0.992 ^d^ |
| Body mass index, mean (SD) ^e^ | 21.06(3.48) | 21.00(3.74) | 21.11(3.43) | 20.82(3.53) | 1-Way ANOVA | 0.032 | 0.726, ^b^ 0.564, ^c^ 0.032 ^d^ |

*Note* SD, standard deviation; ANOVA, analysis of variance; MMSE, Mini-Mental State Examination; NA, not applicable

^a^ The Šídák multiple comparison test was used for all post hoc analyses

^b^ Increasing low sleep quality vs Stable high sleep quality.

^c^ Increasing low sleep quality vs Decreasing sleep quality

^d^ Stable high sleep quality vs Decreasing sleep quality

^e^ Calculated as weight in kilograms divided by height in meters squared

**Table 2.3.** Baseline Characteristics of Participants in the Study (Stratified by Age)

| **Characteristic** | **Total (*n*=8724)** | **Participants, No. (%)** | | | | | |
| --- | --- | --- | --- | --- | --- | --- | --- |
|  |  | **Aged 65-74 y**  **(*n*=2875)** | **Aged 75-84 y**  **(*n*=3621)** | **Aged ≥85 y**  **(*n*=2228)** | **Statistical test** | ***p*-value** | ***p*-value for post hoc test** ^a^ |
| Age, mean (SD), y | 79.31(8.66) | 70.57(2.67) | 78.90(2.79) | 91.24(5.50) | 1-Way ANOVA | <0.001 | <0.001 ^b, c, d^ |
| Sex |  |  |  |  |  |  |  |
| Female | 4334(49.68) | 1446(50.30) | 1830(50.54) | 1058(47.49) | χ^2^ | 0.055 | NA |
| Male | 4390(50.32) | 1429(49.70) | 1791(49.46) | 1170(52.51) |  |  |  |
| District |  |  |  |  |  |  |  |
| Eastern China | 4984(57.13) | 1594(55.44) | 2061(56.92) | 1329(59.65) | χ^2^ | <0.001 | NA |
| Central China | 2253(25.83) | 817(28.42) | 936(25.85) | 500(22.44) |  |  |  |
| Western China | 1487(17.04) | 464(16.14) | 624(17.23) | 399(17.91) |  |  |  |
| Marital status |  |  |  |  |  |  |  |
| Married | 4483(51.39) | 2042(71.03) | 1902(52.53) | 539(24.19) | χ^2^ | <0.001 | NA |
| Divorced | 32(0.37) | 8(0.28) | 18(0.50) | 6(0.27) |  |  |  |
| Widowed | 4135(47.4) | 801(27.86) | 1669(46.09) | 1665(74.73) |  |  |  |
| Never married | 74(0.85) | 24(0.83) | 32(0.88) | 18(0.81) |  |  |  |
| Residence type |  |  |  |  |  |  |  |
| City | 1425(16.33) | 431(14.99) | 591(16.32) | 403(18.09) | χ^2^ | <0.001 | NA |
| Town | 2552(29.25) | 724(25.18) | 1107(30.57) | 721(32.36) |  |  |  |
| Rural | 4747(54.41) | 1720(59.83) | 1923(53.11) | 1104(49.55) |  |  |  |
| Main occupation before 60 y |  |  |  |  |  |  |  |
| Non-manual workers | 888(10.18) | 294(10.23) | 408(11.27) | 186(8.35) | χ^2^ | <0.001 | NA |
| Manual workers | 6992(80.15) | 2350(81.74) | 2888(79.76) | 1754(78.73) |  |  |  |
| Other | 844(9.67) | 231(8.03) | 325(8.98) | 288(12.93) |  |  |  |
| Smoking status |  |  |  |  |  |  |  |
| Present | 1967(22.55) | 777(27.03) | 795(21.96) | 395(17.73) | χ^2^ | <0.001 | NA |
| Previous | 1529(17.53) | 441(15.34) | 684(18.89) | 404(18.13) |  |  |  |
| None | 5228(59.93) | 1657(57.64) | 2142(59.15) | 1429(64.14) |  |  |  |
| Drinking status |  |  |  |  |  |  |  |
| Present | 1889(21.65) | 696(24.21) | 738(20.38) | 455(20.42) | χ^2^ | <0.001 | NA |
| Previous | 1323(15.17) | 393(13.67) | 585(16.16) | 345(15.48) |  |  |  |
| None | 5512(63.18) | 1786(62.12) | 2298(63.46) | 1428(64.10) |  |  |  |
| Exercising status |  |  |  |  |  |  |  |
| Present | 3489(39.99) | 1176(40.90) | 1515(41.84) | 798(35.82) | χ^2^ | <0.001 | NA |
| Previous | 978(11.21) | 276(9.60) | 368(10.16) | 334(14.99) |  |  |  |
| None | 4257(48.8) | 1423(49.50) | 1738(48) | 1096(49.19) |  |  |  |
| Social activities |  |  |  |  |  |  |  |
| Yes | 1690(19.37) | 621(21.60) | 770(21.26) | 299(13.42) | χ^2^ | <0.001 | NA |
| No | 7034(80.63) | 2254(78.40) | 2851(78.74) | 1929(86.58) |  |  |  |
| Sleep parameters |  |  |  |  |  |  |  |
| Sleep duration per night, mean (SD), h | 7.58(2.17) | 7.42(1.87) | 7.46(2.17) | 7.98(2.46) | 1-Way ANOVA | <0.001 | 0.907, ^b^ <0.001 ^c, d^ |
| Sleep quality |  |  |  |  |  |  |  |
| Increasing low sleep quality | 980(11.23) | 349(12.14) | 465(12.84) | 166(7.45) | χ^2^ | <0.001 | NA |
| Stable high sleep quality | 6661(76.35) | 2181(75.86) | 2681(74.04) | 1799(80.75) |  |  |  |
| Decreasing sleep quality | 1083(12.42) | 345(12) | 475(13.12) | 263(11.80) |  |  |  |
| Fruit and vegetable consumption |  |  |  |  |  |  |  |
| Stable high fruit and vegetable consumption | 1871(21.45) | 688(23.93) | 784(21.65) | 399(17.91) | χ^2^ | <0.001 | NA |
| Stable moderate fruit and vegetable consumption | 6611(75.78) | 2137(74.33) | 2741(75.70) | 1733(77.78) |  |  |  |
| Decreasing low fruit and vegetable consumption | 242(2.77) | 50(1.74) | 96(2.65) | 96(4.31) |  |  |  |
| Meat, fish, egg and bean consumption |  |  |  |  |  |  |  |
| Slowly decreasing | 6411(73.49) | 2115(73.57) | 2629(72.60) | 1667(74.82) | χ^2^ | 0.175 | NA |
| Rapidly decreasing | 2313(26.51) | 760(26.43) | 992(27.40) | 561(25.18) |  |  |  |
| Milk consumption |  |  |  |  |  |  |  |
| Never drink milk | 2146(24.6) | 730(25.39) | 867(23.94) | 549(24.64) | χ^2^ | 0.404 | NA |
| Increasing milk consumption | 6578(75.4) | 2145(74.61) | 2754(76.06) | 1679(75.36) |  |  |  |
| Nuts consumption |  |  |  |  |  |  |  |
| Never eat nuts | 2382(27.3) | 525(18.27) | 967(26.71) | 890(39.95) | χ^2^ | <0.001 | NA |
| Increasing nuts consumption | 6342(72.7) | 2350(81.74) | 2654(73.29) | 1338(60.05) |  |  |  |
| Number of chronic diseases |  |  |  |  |  |  |  |
| 0 | 3733(42.79) | 1294(45.01) | 1395(38.52) | 1044(46.86) | χ^2^ | <0.001 | NA |
| 1 | 2360(27.05) | 777(27.03) | 992(27.40) | 591(26.53) |  |  |  |
| ≥2 | 2631(30.16) | 804(27.97) | 1234(34.08) | 593(26.62) |  |  |  |
| Global Cognitive scores, MMSE, mean (SD) | 20.32(3.24) | 21.34(2.24) | 20.39(3.08) | 18.88(3.96) | 1-Way ANOVA | <0.001 | <0.001 ^b, c, d^ |
| Depression scores, mean (SD) | 6.48(3.49) | 6.25(3.35) | 6.51(3.55) | 6.71(3.58) | 1-Way ANOVA | <0.001 | <0.001 ^b, c, d^ |
| Blood pressure, mean (SD), mm Hg |  |  |  |  |  |  |  |
| Systolic | 135.12(23.03) | 132.70(22.16) | 136.51(24.11) | 135.96(22.08) | 1-Way ANOVA | <0.001 | <0.001, ^b, c^ 0.752 ^d^ |
| Diastolic | 80.97(17.31) | 81.61(17.17) | 81.10(18.89) | 79.95(14.54) |  | 0.003 | 0.557, ^b^ 0.002, ^c^ 0.040 ^d^ |
| Heart rate (beats/min) | 74.01(15.15) | 73.50(15.56) | 74.27(16.25) | 74.24(12.53) | 1-Way ANOVA | 0.086 | 0.118, ^b^ 0.223, ^c^ 0.853 ^d^ |
| Body mass index, mean (SD) ^e^ | 21.06(3.48) | 21.37(3.40) | 21.32(3.52) | 20.24(3.38) | 1-Way ANOVA | <0.001 | 0.888, ^b^ <0.001 ^c, d^ |

*Note* SD, standard deviation; ANOVA, analysis of variance; MMSE, Mini-Mental State Examination; NA, not applicable

^a^ The Šídák multiple comparison test was used for all post hoc analyses

^b^ Participants aged 65-74 y vs aged 75-84 y

^c^ Participants aged 65-74 y vs aged ≥85 y

^d^ Participants aged 75-84y vs aged ≥85 y

^e^ Calculated as weight in kilograms divided by height in meters squared

**Table 2.4.** Association between Sleep Duration, Sleep Quality, Food Consumption, and Global Cognitive Scores using Generalized Estimating Equations

|  | **Global cognitive scores** | | | |
| --- | --- | --- | --- | --- |
|  | **RR** ^a^ **(95%CI)** | ***p-*value** | **RR** ^b^ **(95%CI)** | ***p-*value** |
| Sleep duration per night, hours |  |  |  |  |
| Increasing and long sleep duration | -0.023 (-0.146 to 0.100) | 0.711 | -0.051(-0.173 to 0.071) | 0.405 |
| Stable adequate sleep duration | 1[Reference] | NA | 1[Reference] | NA |
| Decreasing and short sleep duration | 0.007 (-0.034 to 0.048) | 0.741 | 0.036(-0.005 to 0.077) | 0.086 |
| Sleep quality |  |  |  |  |
| Increasing low sleep quality | -0.058 (-0.132 to 0.016) | 0.125 | -0.045(-0.118 to 0.028) | 0.112 |
| Stable high sleep quality | 0.011(-0.044 to 0.066) | 0.705 | 0.023(-0.032 to 0.078) | 0.356 |
| Decreasing sleep quality | 1[Reference] | NA | 1[Reference] | NA |
| Fruit and vegetable consumption |  |  |  |  |
| Stable high fruit and vegetable consumption | 0.160(0.121 to 0.199) | <0.001 | 0.088(0.049 to 0.127) | <0.001 |
| Stable moderate fruit and vegetable consumption | 1[Reference] | NA | 1[Reference] | NA |
| Decreasing low fruit and vegetable consumption | -0.170(-0.299 to -0.041) | 0.010 | -0.141(-0.268 to -0.014) | 0.029 |
| Meat, fish, egg and bean consumption |  |  |  |  |
| Slowly decreasing | 1[Reference] | NA | 1[Reference] | NA |
| Rapidly decreasing | -0.165(-0.210 to -0.120) | <0.001 | -0.112(-0.155 to -0.069) | <0.001 |
| Milk consumption |  |  |  |  |
| Never drink milk | 1[Reference] | NA | 1[Reference] | NA |
| Increasing milk consumption | 0.089 (0.046 to 0.132) | <0.001 | 0.044(0.011 to 0.087) | 0.038 |
| Nuts consumption |  |  |  |  |
| Never eat nuts | 1[Reference] | NA | 1[Reference] | NA |
| Increasing nuts consumption | 0.125 (0.080 to 0.170) | <0.001 | 0.082(0.037 to 0.127) | <0.001 |

*Note* CI, confidence interval; NA, not applicable

^a^ After adjusting for age, sex, district, residency type, main occupational, current marriage status

^b^ After adjusting for age, sex, district, residency type, main occupation, current marriage status, smoking status, drinking status, exercise status, frequency of social activities, BMI, depression, chronic diseases, systolic blood pressure, diastolic blood pressure and heart rate

**Table 2.5.** Mediation Analysis of Fruit and Vegetable Consumption in the Association Between Sleep Duration, Sleep Quality and Global Cognitive Scores

| **Mediation Model** | **Standardized model** results | | | | | | | **Model fit parameter** | | | |
| --- | --- | --- | --- | --- | --- | --- | --- | --- | --- | --- | --- |
|  | **Stable high fruit and vegetables consumption** | | | **Stable moderate fruit and vegetables consumption** | **Decreasing low fruit and vegetables consumption** | | |  |  |  |  |
|  | **Estimate** | **95%CI** | ***p***-value |  | **Estimate** | **95%CI** | ***p***-value | **CFI** | **TLI** | **RMSEA** | **SRMR** |
| **Increasing and long sleep duration－**fruit and vegetable consumption ^a^ |  |  |  |  |  |  |  |  |  |  |  |
| Fruit and vegetable consumption on sleep duration | 0.005 | (-0.021 to 0.020) | 0.976 | 1[Reference] | 0.076 | (0.040 to 0.117) | <0.001 | 0.985 | 0.95 | 0.059 | 0.008 |
| Cognitive scores on fruit and vegetable consumption | 0.041 | (0.022 to 0.059) | <0.001 | 1[Reference] | -0.027 | (-0.051 to -0.004) | 0.033 |  |  |  |  |
| Cognitive scores on sleep duration | -0.009 | (-0.034 to 0.014) | 0.447 | 1[Reference] | -0.009 | (-0.034 to 0.014) | 0.447 |  |  |  |  |
| Mediation effects | 0.001 | (-0.004 to 0.004) | 0.977 | 1[Reference] | -0.010 | (-0.024 to -0.002) | 0.043 |  |  |  |  |
| **Decreasing and short sleep duration－**fruit and vegetable consumption ^b^ |  |  |  |  |  |  |  |  |  |  |  |
| Fruit and vegetable consumption on sleep duration | 0.002 | (-0.019 to 0.022) | 0.865 | 1[Reference] | 0.024 | (0.003 to 0.048) | 0.034 |  |  |  |  |
| Cognitive scores on fruit and vegetable consumption | 0.041 | (0.022 to 0.059) | <0.001 | 1[Reference] | -0.027 | (-0.051 to -0.004) | 0.026 |  |  |  |  |
| Cognitive scores on sleep duration | 0.018 | (0.005 to 0.036) | 0.045 | 1[Reference] | 0.018 | (0.005 to 0.036) | 0.045 |  |  |  |  |
| Mediation effects | 0.001 | (-0.002 to 0.002) | 0.868 | 1[Reference] | -0.001 | (-0.006 to -0.001) | 0.145 |  |  |  |  |
| **Increasing low sleep quality－**fruit and vegetable consumption ^c^ |  |  |  |  |  |  |  |  |  |  |  |
| Fruit and vegetable consumption on sleep quality | -0.040 | (-0.058 to -0.021) | <0.001 | 1[Reference] | 0.041 | (0.016 to 0.067) | 0.002 | 0.986 | 0.959 | 0.067 | 0.008 |
| Cognitive scores on fruit and vegetable consumption | 0.041 | (0.022 to 0.059) | <0.001 | 1[Reference] | -0.027 | (-0.051 to -0.004) | 0.028 |  |  |  |  |
| Cognitive scores on sleep quality | 0.001 | (-0.021 to 0.019) | 0.959 | 1[Reference] | 0.001 | (-0.021 to 0.019) | 0.959 |  |  |  |  |
| Mediation effects | -0.005 | (-0.008 to -0.002) | 0.003 | 1[Reference] | -0.003 | (-0.008 to -0.001) | 0.087 |  |  |  |  |
| **Stable high sleep quality－**fruit and vegetable consumption ^d^ |  |  |  |  |  |  |  |  |  |  |  |
| Fruit and vegetable consumption on sleep quality | 0.039 | (0.020 to 0.058) | <0.001 | 1[Reference] | -0.057 | (-0.083 to -0.034) | <0.001 |  |  |  |  |
| Cognitive scores on fruit and vegetable consumption | 0.041 | (0.022 to 0.059) | <0.001 | 1[Reference] | -0.027 | (-0.051 to -0.004) | 0.024 |  |  |  |  |
| Cognitive scores on sleep quality | -0.015 | (-0.035 to 0.006) | 0.153 | 1[Reference] | -0.015 | (-0.035 to 0.006) | 0.153 |  |  |  |  |
| Mediation effects | 0.003 | (0.001 to 0.006) | 0.004 | 1[Reference] | 0.003 | (0.001 to 0.007) | 0.057 |  |  |  |  |

*Note* CFI, comparative fit index; TLI, Tucker-Lewis index; RMSEA, root mean square error of approximation; SRMR, standardized root mean square residual

^a^ Increasing and long sleep duration vs stable adequate sleep duration

^b^ Decreasing and short sleep duration vs stable adequate sleep duration

^c^ Increasing low sleep quality vs decreasing sleep quality

^d^ Stable high sleep quality vs decreasing sleep quality

**Table 2.6.** Mediation Analysis of Meat, Fish, Egg, and Bean Consumption in the Association Between Sleep Duration, Sleep Quality and Global Cognitive Scores

| **Mediation Model** | **Standardized model results** | | | | **Model fit parameter** | | | |
| --- | --- | --- | --- | --- | --- | --- | --- | --- |
|  | **Rapidly decreasing meat, fish, egg, and bean consumption** | | | **Slowly decreasing meat, fish, egg, and bean consumption** |  |  |  |  |
|  | **Estimate** | **95%CI** | ***p*-value** |  | **CFI** | **TLI** | **RMSEA** | **SRMR** |
| **Increasing and long sleep duration－meat, fish, egg, and bean consumption** ^a^ |  |  |  |  |  |  |  |  |
| Meat, fish, egg, and bean consumption on sleep duration | 0.017 | (-0.005 to 0.039) | 0.133 | 1[Reference] | 1.000 | 1.000 | <0.001 | <0.001 |
| Cognitive scores on meat, fish, egg, and bean consumption | -0.055 | (-0.076 to -0.034) | <0.001 | 1[Reference] |  |  |  |  |
| Cognitive scores on sleep duration | -0.010 | (-0.034 to 0.012) | 0.399 | 1[Reference] |  |  |  |  |
| Mediation effects | -0.005 | (-0.012 to 0.001) | 0.155 | 1[Reference] |  |  |  |  |
| **Decreasing and short sleep duration－meat, fish, egg, and bean consumption** ^b^ |  |  |  |  |  |  |  |  |
| Meat, fish, egg, and bean consumption on sleep duration | 0.072 | (0.051 to 0.092) | <0.001 | 1[Reference] |  |  |  |  |
| Cognitive scores on meat, fish, egg, and bean consumption | -0.055 | (-0.076 to -0.034) | <0.001 | 1[Reference] |  |  |  |  |
| Cognitive scores on sleep duration | 0.022 | (0.003 to 0.040) | 0.022 | 1[Reference] |  |  |  |  |
| Mediation effects | -0.009 | (-0.014 to -0.005) | <0.001 | 1[Reference] |  |  |  |  |
| **Increasing low sleep quality－meat, fish, egg, and bean consumption** ^c^ |  |  |  |  |  |  |  |  |
| Meat, fish, egg, and bean consumption on sleep quality | 0.097 | (0.075 to 0.120) | <0.001 | 1[Reference] | 1.000 | 1.000 | <0.001 | <0.001 |
| Cognitive scores on meat, fish, egg, and bean consumption | -0.055 | (-0.076 to -0.034) | <0.001 | 1[Reference] |  |  |  |  |
| Cognitive scores on sleep quality | 0.003 | (-0.018 to 0.021) | 0.750 | 1[Reference] |  |  |  |  |
| Mediation effects | -0.015 | (-0.023 to -0.009) | <0.001 | 1[Reference] |  |  |  |  |
| **Stable high sleep quality－meat, fish, egg, and bean consumption** ^d^ |  |  |  |  |  |  |  |  |
| Meat, fish, egg, and bean consumption on sleep quality | -0.011 | (-0.133 to -0.088) | <0.001 | 1[Reference] |  |  |  |  |
| Cognitive scores on meat, fish, egg, and bean consumption | -0.055 | (-0.076 to -0.034) | <0.001 | 1[Reference] |  |  |  |  |
| Cognitive scores on sleep quality | -0.018 | (-0.038 to 0.002) | 0.081 | 1[Reference] |  |  |  |  |
| Mediation effects | 0.013 | (0.008 to 0.020) | <0.001 | 1[Reference] |  |  |  |  |

*Note* CFI, comparative fit index; TLI, Tucker-Lewis index; RMSEA, root mean square error of approximation; SRMR, standardized root mean square residual

^a^ Increasing and long sleep duration vs stable adequate sleep duration

^b^ Decreasing and short sleep duration vs stable adequate sleep duration

^c^ Increasing low sleep quality vs decreasing sleep quality

^d^ Stable high sleep quality vs decreasing sleep quality

**Table 2.7.** Mediation Analysis of Milk Consumption in the Association Between Sleep Duration, Sleep Quality and Global Cognitive Scores

| **Mediation Model** | **Standardized model results** | | | | **Model fit parameter** | | | |
| --- | --- | --- | --- | --- | --- | --- | --- | --- |
|  | **Increasing milk consumption** | | | **Never drink milk** |  |  |  |  |
|  | **Estimate** | **95%CI** | ***p*-value** |  | **CFI** | **TLI** | **RMSEA** | **SRMR** |
| **Increasing and long sleep duration－milk consumption** ^a^ |  |  |  |  |  |  |  |  |
| Milk consumption on sleep duration | 0.011 | (-0.010 to 0.030) | 0.278 | 1[Reference] | 1.000 | 1.000 | <0.001 | <0.001 |
| Cognitive scores on milk consumption | 0.021 | (0.005 to 0.041) | 0.047 | 1[Reference] |  |  |  |  |
| Cognitive scores on sleep duration | -0.011 | (-0.036 to 0.012) | 0.346 | 1[Reference] |  |  |  |  |
| Mediation effects | 0.001 | (-0.001 to 0.005) | 0.392 | 1[Reference] |  |  |  |  |
| **Decreasing and short sleep duration－milk consumption** ^b^ |  |  |  |  |  |  |  |  |
| Milk consumption on sleep duration | 0.030 | (0.010 to 0.049) | 0.003 | 1[Reference] |  |  |  |  |
| Cognitive scores on milk consumption | 0.021 | (0.005 to 0.041) | 0.047 | 1[Reference] |  |  |  |  |
| Cognitive scores on sleep duration | 0.017 | (-0.002 to 0.035) | 0.073 | 1[Reference] |  |  |  |  |
| Mediation effects | 0.001 | (-0.002 to 0.003) | 0.120 | 1[Reference] |  |  |  |  |
| **Increasing low sleep quality－milk consumption** ^c^ |  |  |  |  |  |  |  |  |
| Milk consumption on sleep quality | 0.009 | (-0.012 to 0.027) | 0.396 | 1[Reference] | 1.000 | 1.000 | <0.001 | <0.001 |
| Cognitive scores on milk consumption | 0.021 | (0.005 to 0.041) | 0.047 | 1[Reference] |  |  |  |  |
| Cognitive scores on sleep quality | -0.002 | (-0.024 to 0.016) | 0.813 | 1[Reference] |  |  |  |  |
| Mediation effects | 0.001 | (-0.001 to 0.002) | 0.478 | 1[Reference] |  |  |  |  |
| **Stable high sleep quality－milk consumption** ^d^ |  |  |  |  |  |  |  |  |
| Milk consumption on sleep quality | 0.022 | (0.001 to 0.043) | 0.042 | 1[Reference] |  |  |  |  |
| Cognitive scores on milk consumption | 0.021 | (0.005 to 0.041) | 0.047 | 1[Reference] |  |  |  |  |
| Cognitive scores on sleep quality | -0.012 | (-0.003 to 0.008) | 0.244 | 1[Reference] |  |  |  |  |
| Mediation effects | 0.001 | (-0.001 to 0.003) | 0.185 | 1[Reference] |  |  |  |  |

*Note* CFI, comparative fit index; TLI, Tucker-Lewis index; RMSEA, root mean square error of approximation; SRMR, standardized root mean square residual

^a^ Increasing and long sleep duration vs stable adequate sleep duration

^b^ Decreasing and short sleep duration vs stable adequate sleep duration

^c^ Increasing low sleep quality vs decreasing sleep quality

^d^ Stable high sleep quality vs decreasing sleep quality

**Table 2.8.** Mediation Analysis of Nuts Consumption in the Association Between Sleep Duration, Sleep Quality and Global Cognitive Scores

| **Mediation Model** | **Standardized model results** | | | | **Model fit parameter** | | | |
| --- | --- | --- | --- | --- | --- | --- | --- | --- |
|  | **Increasing nuts consumption** | | | **Never eat nuts** |  |  |  |  |
|  | **Estimate** | **95%CI** | ***p*-value** |  | **CFI** | **TLI** | **RMSEA** | **SRMR** |
| **Increasing and long sleep duration－nuts consumption** ^a^ |  |  |  |  |  |  |  |  |
| Nuts consumption on sleep duration | -0.022 | (-0.044 to 0.001) | 0.062 | 1[Reference] | 1.000 | 1.000 | <0.001 | <0.001 |
| Cognitive scores on nuts consumption | 0.041 | (0.019 to 0.063) | <0.001 | 1[Reference] |  |  |  |  |
| Cognitive scores on sleep duration | -0.010 | (-0.035 to 0.013) | 0.396 | 1[Reference] |  |  |  |  |
| Mediation effects | -0.005 | (-0.011 to 0.001) | 0.105 | 1[Reference] |  |  |  |  |
| **Decreasing and short sleep duration－nuts consumption** ^b^ |  |  |  |  |  |  |  |  |
| Nuts consumption on sleep duration | 0.002 | (-0.018 to 0.022) | 0.839 | 1[Reference] |  |  |  |  |
| Cognitive scores on nuts consumption | 0.041 | (0.019 to 0.063) | <0.001 | 1[Reference] |  |  |  |  |
| Cognitive scores on sleep duration | 0.017 | (-0.001 to 0.036) | 0.064 | 1[Reference] |  |  |  |  |
| Mediation effects | 0.002 | (-0.002 to 0.005) | 0.844 | 1[Reference] |  |  |  |  |
| **Increasing low sleep quality－nuts consumption** ^c^ |  |  |  |  |  |  |  |  |
| Nuts consumption on sleep quality | -0.027 | (-0.049 to -0.006) | 0.012 | 1[Reference] | 1.000 | 1.000 | <0.001 | <0.001 |
| Cognitive scores on nuts consumption | 0.041 | (0.019 to 0.063) | <0.001 | 1[Reference] |  |  |  |  |
| Cognitive scores on sleep quality | -0.001 | (-0.023 to 0.017) | 0.915 | 1[Reference] |  |  |  |  |
| Mediation effects | -0.003 | (-0.007 to -0.001) | 0.045 | 1[Reference] |  |  |  |  |
| **Stable high sleep quality－nuts consumption** ^d^ |  |  |  |  |  |  |  |  |
| Nuts consumption on sleep quality | 0.043 | (0.021 to 0.065) | <0.001 | 1[Reference] |  |  |  |  |
| Cognitive scores on nuts consumption | 0.041 | (0.019 to 0.063) | <0.001 | 1[Reference] |  |  |  |  |
| Cognitive scores on sleep quality | -0.013 | (-0.034 to 0.006) | 0.194 | 1[Reference] |  |  |  |  |
| Mediation effects | 0.004 | (0.002 to 0.008) | 0.009 | 1[Reference] |  |  |  |  |

*Note* CFI, comparative fit index; TLI, Tucker-Lewis index; RMSEA, root mean square error of approximation; SRMR, standardized root mean square residual

^a^ Increasing and long sleep duration vs stable adequate sleep duration

^b^ Decreasing and short sleep duration vs stable adequate sleep duration

^c^ Increasing low sleep quality vs decreasing sleep quality

^d^ Stable high sleep quality vs decreasing sleep quality
